# Functional Characterization and Signaling Systems of Corazonin and Red Pigment Concentrating Hormone in the Green Shore Crab, *Carcinus maenas*

**DOI:** 10.3389/fnins.2017.00752

**Published:** 2018-01-15

**Authors:** Jodi L. Alexander, Andrew Oliphant, David C. Wilcockson, Neil Audsley, Rachel E. Down, Rene Lafont, Simon G. Webster

**Affiliations:** ^1^School of Biological Sciences, Brambell Laboratories, Bangor University, Bangor, United Kingdom; ^2^Institute of Biological Environmental and Rural Sciences, Aberystwyth University, Aberystwyth, United Kingdom; ^3^Fera Science Ltd, York, United Kingdom; ^4^IBPS-BIOSIPE, Sorbonne Universités, UPMC Univ Paris 06, Paris, France

**Keywords:** *Carcinus maenas*, neuropeptides, G protein-coupled receptors, corazonin, red pigment concentrating hormone, mRNA and peptide expression, neuroanatomy, physiological roles

## Abstract

Neuropeptides play a central role as neurotransmitters, neuromodulators and hormones in orchestrating arthropod physiology. The post-genomic surge in identified neuropeptides and their putative receptors has not been matched by functional characterization of ligand-receptor pairs. Indeed, until very recently no G protein-coupled receptors (GPCRs) had been functionally defined in any crustacean. Here we explore the structurally-related, functionally-diverse gonadotropin-releasing hormone paralogs, corazonin (CRZ) and red-pigment concentrating hormone (RPCH) and their G-protein coupled receptors (GPCRs) in the crab, *Carcinus maenas*. Using aequorin luminescence to measure *in vitro* Ca^2+^ mobilization we demonstrated receptor-ligand pairings of CRZ and RPCH. CRZR-activated cell signaling in a dose-dependent manner (EC_50_ 0.75 nM) and comparative studies with insect CRZ peptides suggest that the C-terminus of this peptide is important in receptor-ligand interaction. RPCH interacted with RPCHR with extremely high sensitivity (EC_50_ 20 pM). Neither receptor bound GnRH, nor the AKH/CRZ-related peptide. Transcript distributions of both receptors indicate that CRZR expression was, unexpectedly, restricted to the Y-organs (YO). Application of CRZ peptide to YO had no effect on ecdysteroid biosynthesis, excepting a modest stimulation in early post-molt. CRZ had no effect on heart activity, blood glucose levels, lipid mobilization or pigment distribution in chromatophores, a scenario that reflected the distribution of its mRNA. Apart from the well-known activity of RPCH as a chromatophorotropin, it also indirectly elicited hyperglycemia (which was eyestalk-dependent). RPCHR mRNA was also expressed in the ovary, indicating possible roles in reproduction. The anatomy of CRZ and RPCH neurons in the nervous system is described in detail by immunohistochemistry and *in situ* hybridization. Each peptide has extensive but non-overlapping distribution in the CNS, and neuroanatomy suggests that both are possibly released from the post-commissural organs. This study is one of the first to deorphanize a GPCR in a crustacean and to provide evidence for hitherto unknown and diverse functions of these evolutionarily-related neuropeptides.

## Introduction

Cellular signaling, which pervades and integrates all aspects of metazoan life, involves a seemingly bewildering variety of messenger molecules. For neurons, apart from small molecules and gaseous transmitters, the overwhelming majority of such signaling involves a complex interplay of an impressively diverse array of neuropeptides which act as neurotransmitters, neuromodulators or circulating neurohormones. The first step in signal transduction, for the majority of neuropeptides, involves interaction with plasmamembrane bound G protein-coupled receptors (GPCRs). With the advent of large scale genome and transcriptome sequencing and analysis, a huge variety of neuropeptides and their putative cognate GPCRs have been identified, and leading on from the sequencing of the *Drosophila* genome over 15 years ago (Adams et al., 2000), more than 250 genomes have now been sequenced for insects. In contrast, genomic and transcriptomic data on the identity and function of crustacean neuropeptides and cognate receptors has lagged behind those of insects, despite their huge economic importance in aquaculture. Nevertheless, *in silico* genome and transcriptome mining has led to the discovery of a large number of peptide and putative peptide receptors in crustaceans (See Christie et al., 2015 for a comprehensive list). In particular that study identified 35 neuropeptide precursor encoding transcripts (194 predicted peptides), and 41 putative GPCR encoding transcripts in the American lobster, *Homarus americanus*, and this inventory has now been even further expanded (Christie et al., 2017). However, the functional identification of putative GPCR ligands pairs, without rational deorphanisation strategies is, as reiterated by Caers et al. (2012) fraught with difficulty. Indeed, correlating sequence identity with functionality is perhaps meaningless given the inevitable sequence similarities within the five subfamilies of GPCRs; i.e. rhodopsin, secretin, glutamate, adhesion and frizzled-tastes-2 (Fredriksson et al., 2003).

The consensus view of arthropod evolution is one involving a monophyletic ancestor to the crustacean and insect lineages (Cook et al., 2001; Regier et al., 2010) and despite the passage of time (over 400 million years since divergence), it is apparent that many neuropeptide ligand/receptor signaling systems have been conserved. In particular, we are interested in the ones that regulate the most pervasive physiologies of all arthropods-those involved in ecdysis. During a transcriptomic screen of tissue specific and differentially expressed transcripts in the green shore crab, *Carcinus maenas*, we noticed that one candidate GPCR with high similarity to that of the corazonin receptor (CRZR) originally cloned and deorphanised in *Drosophila melanogster* (Cazzamali et al., 2002; Park et al., 2002) and *Anopheles gambiae* (Belmont et al., 2006), was highly expressed by the Y-organ (YO). This was interesting since CRZ signaling is an ancient and conserved system in arthropods. Representatives of this peptide/receptor family also includes the adipokinetic hormones (AKH) of insects and the related red pigment concentrating hormone (RPCH) of crustaceans, and adipokinetic hormone/corazonin-related peptide (ACP). It is believed that evolution of this neuropeptide family, and their cognate GPCRs had an early Urbilaterian origin about 700 million years ago at the split of the Proto- and Deuterostomian lineages via gene duplication from a gonadotropin releasing hormone (GnRH)-like hormone and receptor. The recent important findings that GnRH and CRZ signaling pathways (both peptides and receptors) are found in echinoderms (Tian et al., 2016); are of particular significance in supporting this hypothesis. It is believed that a second gene duplication in a common ancestor to the Arthropoda gave rise to AKH- and ACP-type receptors, whilst CRZ-type receptors have been lost in multiple (vertebrate) lineages (Reviews: Sakai et al., 2017; Zandawala et al., 2017). The evolution, emergence (and loss) of members of the AKH/CRZ/ACP peptide and receptor superfamily in lophotrochozoans and arthropods have been recently reviewed (Hauser and Grimmelikhuijzen, 2014; Li et al., 2016).

Whilst the biological activities of AKHs in insects are related to metabolism: mobilization of energy reserves (reviews Gäde and Auerswald, 2003; Lorenz and Gäde, 2009), and more recently, neuroendocrine regulation of related metabolic processes (Gáliková et al., 2017), CRZ appears to regulate a variety of processes unrelated to its originally described function (and name) which was acceleration of heart rate in the cockroach *Periplaneta americana* (Veenstra, 1989) and *Rhodnius prolixus* (Patel et al., 2014). For example, injection of [His ^7^]-CRZ leads to phase transition associated darkening of the body in locusts (Tawfik et al., 1999; Tanaka, 2000; Roller et al., 2003), and has been shown to be a central regulator of caste identity in ants (Bonasio et al., 2010; Patalano et al., 2015). A recent study has elegantly shown that CRZ supresses vitellogenin gene expression in the brain of *Harpegnathos saltator*, whilst stimulating worker behaviors and inhibiting dueling and egg laying (Gospocic et al., 2017). In the silkworm, *Bombyx mori*, CRZ reduces the spinning rate of silk during the larva-pupa transition (Tanaka et al., 2002). Involvement of CRZ in circadian clockwork has been suggested from colocalization of CRZ immunoreactivity with Period (PER) in *Manduca sexta* (Wise et al., 2002), and PER and Doubletime (DBT) in *B. mori* cerebral ganglia (Qi-Miao et al., 2003). Of particular interest is the observation that CRZ initiates ecdysis behavior by stimulating release of pre-ecdysis triggering hormone (PETH) and ecdysis triggering hormone (ETH) from Inka cells in *M. sexta* larvae (Kim et al., 2004). Additionally, it has recently been shown that in the fruit fly *Bactrocera dorsalis* the CRZ receptor (CRZR) is highly expressed in the epitracheal gland (containing the Inka cells that produce PETH and ETH) and that RNAi of CRZR delays larval- pupal transition and pupariation, the latter being associated with delayed expression of tyrosine hydroxylase and dopa-decarboxylase genes (Hou et al., 2017). Thus, the emerging scenario is one that implicates CRZ signaling in a rather wide variety of functions in arthropods, and it has been suggested that the overarching roles of CRZ might be related to feeding, stress or starvation induced stress (Veenstra, 2009; Boerjan et al., 2010). Recent studies involving knockdown of the CRZ receptor (CRZR) in *Drosophila* clearly point to such roles in regulation of metabolic homeostasis in this insect (Kubrak et al., 2016).

Since our screens of neural, epidermal and YO transcriptomes of *C. maenas* resulted in the identification of a richly diverse and comprehensive inventory of neuropeptides and their putative receptors (Oliphant et al., under review), see also Veenstra (2016), and in particular, since we had identified a putative CRZR which was highly expressed in a tissue fundamental to ecdysis- the YO, and furthermore, since no crustacean GPCRs have, yet been functionally deorphanised, with the recent exception of RPCHR in the water flea, *Daphnia pulex* (Marco et al., 2017), we undertook to answer the question: What are the identities and physiological roles of the corazonin neuropeptide family and associated cognate receptors in our crustacean model, *C. maenas*?

## Materials and methods

### Animals, and tissue collection

Mature green shore crabs, *C. maenas* were collected using baited traps, or by hand from the Menai Strait, UK, maintained in a recirculating seawater system at ambient temperature and photoperiod and fed *ad libitum*. Molt staging was performed according to Drach and Tchernigovtzeff (1967). For RNA extractions, tissues were microscopically dissected from ice-anesthetized animals in ice-cold physiological saline (Webster, 1986), immediately frozen in liquid nitrogen and stored at −80°C. For immunohistochemistry (IHC) or *in situ* hybridization (ISH), eyestalk neural tissues, cerebral and ventral ganglia were dissected and fixed in Stephanini's fixative (Stephanini et al., 1967) for IHC or 4% paraformaldehyde (PFA) in phosphate buffered saline for ISH, overnight at 4°C.

### Transcriptome sequencing and assembly

Transcriptome sequencing of neural, YO, and epidermis tissue was performed as described elsewhere (Oliphant et al., under review). Briefly, cDNA libraries were prepared from total RNA using Illumina TruSeq RNA sample preparation reagents and sequenced as paired end reads (126 bp) across multiple lanes on an Illumina HiSeq 2500 platform. Raw reads were head-cropped by 13 bp before *de novo* assembly using Trinity v2.0.6 with “trimmomatic” and “normalize read” options enabled (Grabherr et al., 2011; Bolger et al., 2014; MacManes, 2014). Transcriptomes for neural and Y-organ tissues, and a third comprising YO and epidermis tissues, were assembled separately. Differential gene expression analysis between YO and epidermis tissues was done using Corset (Davidson and Oshlack, 2014) and edgeR (Robinson et al., 2010) to identify GPCRs upregulated in the YO relative to the epidermis. Transcriptomes were mined for contigs putatively encoding neuropeptide receptors using tBLASTn local searches in BioEdit software (Hall, 1999). Neuropeptide and GPCR protein sequences used as search terms were taken from the NCBI database. Contigs mined as putative neuropeptide receptor sequences were translated using the online tool ExPASy Translate (http://web.expasy.org/translate/, Artimo et al., 2012), submitted to tBLASTn searches against the NCBI database, and transmembrane helix domains predicted using TMHMM server v2.0 (http://www.cbs.dtu.dk/services/TMHMM/, Sonnhammer et al., 1998).

### Quantitative RT-PCR

Gene expression was performed using Taqman MGB hydrolysis probes as described previously (Hoelters et al., 2016) using standard curves made from *in vitro* transcribed cRNA. In brief, standards were made from PCR products generated using gene-specific primers 5′ flanked by T7 phage promoter sequences. Transcription reactions were done using Megashortscript reagents (Invitrogen, Paisley, UK) and products gel purified on 10% 6M urea PAGE gels and eluted overnight at room temperature in “Elution buffer” according to manufacturer's instructions (Ambion). Complementary RNA yield was quantified using Avogadro's constant and converted to copy number per qPCR reaction. Standards and 1 μg DNAse-treated (Turbo DNAse, Invitrogen) sample RNA were reverse transcribed in 10 μl reactions using anchored oligo dT primers and Tetro cDNA synthesis reagents (Bioline, UK) according to the manufacturer's instructions. Standard curves were run in the range 18^8^-10^2^ copies. Duplex qPCR reactions (10 μl) amplifying the target gene and reference genes were done in triplicate with Bioline Sensifast Probe II qPCR reagents (Bioline) and run on an Applied Biosystems QuantStudio 12-Flex machine. Data were expressed as copies of target cDNA normalized to the geometric mean of reference gene copies. The reference genes, *elongation factor-1* and *Ubiquitin-conjugating enzyme E2 L3*, were chosen based on their stable expression over all molt stages, as determined by RNAseq analyses.

### Isolation and cloning cDNA encoding CRZR, RPCHR: receptor assays

#### RNA extraction and cDNA synthesis

Total RNA was extracted from frozen tissues using TRIzol (Invitrogen, Carlsbad, USA) according to the manufacturer's instructions, followed by removal of gDNA with DNase 1 (TURBO DNA-free kit, Invitrogen). mRNA was purified using Dynabeads Oligo (dT)_25_ (Dynal, Oslo, Norway) and stored in 10 mM Tris-HCl at −80°C. First strand cDNA synthesis was carried out using Tetro cDNA synthesis kit (Bioline, UK) using a mix of random hexamers and oligo(dT)_18_ primers and the following conditions: 25°C 10 min, 45°C 30 min, 85°C for 5 min.

### Receptor cloning

As described in Oliphant et al. (under review), transcriptome data were queried for GPCR receptor sequences differentially expressed between epidermal and YO tissues. Sequences highly expressed in YO and neural tissue were targeted for further studies. Additionally, the ACP receptor (Hansen et al., 2010) and RPCH receptor sequences (Staubli et al., 2002) were used to identify candidate sequences in the *C. maenas* transcriptome.

Gene-specific primers for each receptor sequence (Invitrogen, Paisley, UK) were designed to be 21–27 nt, 40–70% GC content and a melting temperature of 60–62°C. Sequences are given in Supplementary Table 1. The targets were amplified using Phusion® High Fidelity DNA Polymerase (New England BioLabs, Ipswich, MA) in 25 μl reactions with the following conditions: 98°C for 7 min; 35 cycles of 98°C 30 s, 65–72°C 30 s, 72°C 1:45 min; then 72°C for 10 min. PCR products were electrophoresed on 2% agarose gels and the appropriate bands excised and purified using an Agarose GelExtract Mini Kit (5Prime, Hamburg). Positive amplicons were directionally sub-cloned into pcDNA™ 3.1 D/V5-His-TOPO plasmids and the recombinant vector transformed into One Shot® TOP10 Competent cells (Invitrogen, Paisley, UK). Positive clones were cultured overnight in LB with 100 μg ml^−1^ ampicillin and the plasmids extracted using a FastPlasmid Mini Kit (5Prime, Hamburg) as directed. Purified plasmids were sequenced (MWG Eurofins, Ebersberg, Germany) and analysed using Geneious version 9.1.8 (Kearse et al., 2012).

### Cell culture and receptor assays

Chinese hamster ovary (CHO-K1) cells containing stably expressed apoaequorin (Perkin Elmer, Boston, MA) and either Gα16 or G_*q*_ subunit (control cells) were cultured in Dulbecco's Modified Eagle Medium (DMEM) F-12 Nutrient Mixture GlutaMax (Gibco®) supplemented with 10% fetal bovine serum (Gibco®). Cells were maintained in vented T75 flasks at 37°C in 5% CO_2_.

Cells grown in a monolayer to approximately 60% confluency in T75 flasks were transfected with pcDNA3.1 constructs using FugeneHD (Promega) according to the manufacturer's recommendations. Briefly, transfection medium was prepared by combining 800 μl Opti-MEM® (Gibco) with 10 μg vector and 30 μl Fugene HD in a 2 ml polystyrene tube and incubated for 10 min at room temperature. The transfection reagent was added to 5 ml of fresh culture media and the cells incubated overnight at 37°C in 5% CO_2_. After 24 h, an additional 10 ml of culture media were added to the flasks and the cells returned to 37°C at 5% CO_2_ for a further overnight incubation.

On the day of the assay, cells were detached from the culture flask by incubating for 12 min in 5 ml 0.2% EDTA in PBS. The cells were washed with 10 ml DMEM clear DMEM/F-12 containing L-glutamine, 15 mM HEPES and moved to 50 ml centrifuge tubes. Cells were centrifuged for 5 min at 260 *g* and resuspended to a concentration of 5 × 10^6^ cells ml^−1^ in 0.2 μm filtered BSA medium (DMEM/F-12 containing L-glutamine, 15 mM HEPES + 0.1% BSA). Coelenterazine *h* (Invitrogen, Paisley, UK) was added to a concentration of 5 μM and the cells incubated for 4 h in the dark at room temperature whilst gently rocking. The cells were diluted 10-fold and incubated for a further 60 min before use in the assay. Synthetic peptides (GeneCust, Dudelange, Luxembourg) were reconstituted in 30% acetonitrile, dried in small aliquots by vacuum centrifugation and subsequently redissolved in BSA medium. Fifty microliter aliquots were then dispensed into quadruplicate wells of a white 96-well plate (OptiPlate, PerkinElmer). Cell suspensions were gently stirred and injected in 50 μl aliquots into each well using a Mithras LB 940 microplate reader (Berthold Technologies, Bad-Wildbad, Germany) and light emission (Ca^2+^ response) recorded for 30 s. Cells were then lysed by injection of 0.3% Triton-X in BSA medium and light emission recorded for a further 10 s to measure total Ca^2+^ response. BSA medium was used for blank measurements (6 replicate wells per plate) and mock transfection with empty vectors carried out for negative controls. Data was analyzed using MikroWin v5.18 (Mikrotek Laborsysteme GmbH) and SigmaPlot v.13 (Systat Software, Inc.). Receptor response was normalized against total Ca^2+^ response.

### Immunohistochemistry and *in situ* hybridization

Antisera were raised commercially in rabbits to synthetic crab/insect CRZ (pQTFQYSRGWTN-NH_2_) and *C. maenas* ACP (pQITFSRSWVPQ-NH_2)_, conjugated to bovine thyroglobulin, and sera were purified by affinity chromatography with immobilized ligand (Davids Biotechnologie, Ulm, Germany). An antiserum to RPCH (pQLNFSPGW-NH_2_) was raised in-house (Dircksen and Chung, unpublished). Specificity controls were done by preabsorbtion of 100-fold molar excess ligand to antiserum and by incubating with preimmune sera and processing for IHC as detailed below. In no case was non-specific immunolabelling observed.

Fixed nervous systems were processed for whole mount IHC according to Webster et al. (2012). Primary antiserum dilutions were: anti-CRZ 1:1-3000, anti-RPCH 1:1000, anti-ACP 1:100. Secondary antiserum dilution (Alexa Fluor 488 goat anti-rabbit, Invitrogen, Thermo Fisher Scientific) was 1:750. Preparations were mounted on cavity microscope slides with Vectashield (Vectorlabs, UK), coverslipped, sealed with nail varnish, and images were collected and Z-stacked (5 μm intervals) on a Zeiss 710 confocal microscope equipped with Zen 10 software (Carl Zeiss AG, Jena, Germany).

*In-situ* hybridization was done using digoxygenin-labeled riboprobes as previously described (Wilcockson et al., 2011). Probe synthesis was performed using primers detailed in Supplementary Table 1. Preparations were mounted in 50% glycerol/PBS, sealed as described, and 3D stacks of several planes of focus were imaged using Helicon Focus 6 (HeliconSoft, Karkiv, Ukraine). Images were cropped, resized and adjusted for brightness and contrast using Adobe Photoshop CC 2017 and CorelDraw 2014.

### Bioassays

Y-organ (YO) bioassays to measure the activity of CRZ (50 nM) on ecdysteroid synthesis were performed on YO pairs from molt-staged crabs as previously described (Chung and Webster, 1996). *C. maenas* MIH, prepared and quantified according to Webster (1991) was used as a positive control (10 nM). Secreted ecdysteroids were measured by radioimmunoassay (RIA) using the H1 antiserum (1:1000) (W.E. Bollenbacher, a kind gift from E.S. Chang, U.C. Davis, Bodega Bay Laboratory) which cross-reacts equally with 25-deoxyecdysone and ecdysone. ^3^H ecdysone (NEN, Boston, USA), specific activity *ca*. 1TBq mmol^−1^ (30,000 dpm/tube), was used as radioligand and ecdysone 5000-19pg/tube as standards. Bound radioligand was separated using Sac-Cel immobilized Donkey anti-rabbit IgG (Immuno Diagnostic Services, Tyne and Wear, UK).

Ecdysteroids secreted in bioassays by YO pairs from intermolt (C4) and premolt (D2) crabs. Treated YO and treated YO were cultured in CRZ (50 nM) and analyzed by LC-MS. Culture media were dried in a vacuum centrifuge and then taken up in 60 μl methanol including 3 μl internal standard (Polypodine B, 10 μg/ml). The samples were separated on a LC 1260 Infinity coupled with a 6420 Triple Quadrupole Mass Spectrometer (Agilent Technologies) with multiple reaction monitoring (MRM) in the positive ion mode. Chromatographic conditions: Column: Fortis C_18_, 2.5 μm 2.1 × 50 mm, Solvents and gradient: 10–60% acetonitrile containing 0.1% formic acid 0–7 min, 60–100% 7–9 min, 0.3 ml/min. Standard ecdysteroids (5 mg/ml in DMSO) were 25-deoxyecdysone, ecdysone and 3-dehydroecdysone.

Pigmentary effector activity of CRZ, RPCH, *Locusta migratoria* AKH-I, and ACP were measured *in vivo*, using small (15–20 mm carapace width) *C. maenas*. Crabs were firstly adapted to a white background, light intensity 300 lx for 1 h. Pigment dispersion of red and black chromatophores in the dactyl of the 5th walking leg were microscopically observed and indices of dispersion scored according to Hogben and Slome (1931). Red and black chromatophores were firstly fully dispersed by injection of 5 pmol *C. maenas* pigment dispersing hormone (NSELINSILGLPKVMNDA-NH_2_) in 10 μl saline into the hypobranchial sinus, using hand-drawn glass microcapillaries. Chromatophore dispersion indices were scored at 0, 20, 40 min. Peptides were then injected and chromatophores subsequently scored after 5 and 20 min.

Adipokinetic and glucose mobilizing activity of CRZ, RPCH and ACP were performed by measurement of triglycerides in hemolymph (Tennessen et al., 2014) and glucose (Chung and Webster, 1996). Hemolymph samples (*ca*. 100 μl) were taken from crabs injected with each peptide at 0 and 90 min, and subsequently assayed.

Cardioactivity of CRZ, RPCH, and ACP was performed on semi-isolated heart preparations of *C. maenas*. Crabs (*ca*. 65 mm carapace width) were ice-anesthetized and rapidly decerebrated before removing all limbs and the dorsal carapace to expose the heart and pericardial cavity. The heart was connected to a force transducer (MLT0210/A) via a micro fishing hook (size 28) and fine nylon (0.08 mm) monofilament. Connection to a PC with Chart 4.0 was via a Bridge Pod (ML301) and Powerlab 4/20 (AD Instruments Pty Ltd, Castle Hill, NSW, Australia). Transducer gain was set at maximum sensitivity (200 μV). Heart preparations were initially perfused with *C. maenas* physiological saline (Saver et al., 1999) at room temperature (20°C). Once a stable heart output was achieved, hearts were firstly perfused with 1 ml saline, followed by increasing concentrations of peptides, applied in 1 ml. Crustacean cardioactive peptide (CCAP) (Bachem, Bubendorf, Switzerland), or proctolin (Sigma, Poole, UK) were positive controls (10^−11^-10^−7^ M). Test peptides (CRZ, RPCH, ACP) were perfused at 10^−8^-10^−6^ M for approximately 2 min at each concentration.

## Results

### CRZR and RPCHR analysis

**Figure 1:**
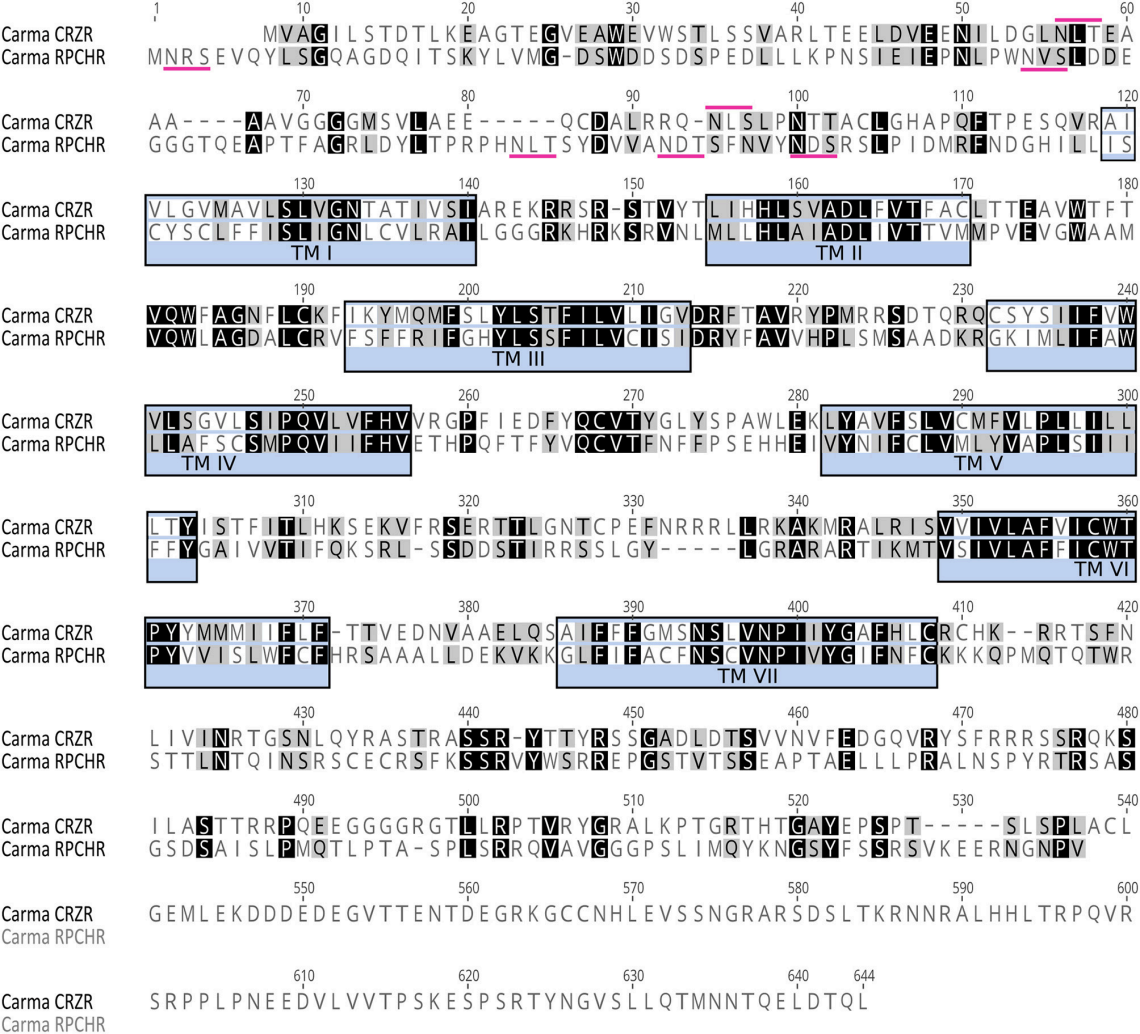
Amino acid sequence analysis of Carma-CRZR (Accession No. MF974386) and Carma-RPCHR (Accession No.MF974387). Amino acid similarity is indicated by color: identical residues are highlighted in black and conservatively substituted residues in gray. The seven transmembrane regions are identified by blue boxes. Predicted N-glycosylation sites in the N-terminus are identified by magenta markers.

The complete cDNA sequences encoding GPCRs were identified from tBLASTn searches of the *Carcinus* transcriptomes (TSA accession code: GFX F00000000, BioProject No. PRJNA400568, Oliphant et al., under review) and functionally identified as the corazonin receptor (CRZR) and red pigment concentrating hormone receptor (RPCHR) (Accession Nos. MF974386, MF974387 respectively). Likewise, full length cDNA sequences encoding CRZ, RPCH and ACP were identified (Accession Nos: MF977831, S65357, MF977832 respectively). Deduced amino acid sequences for CRZR and RPCHR are shown on Figure 1. Full length sequences encoding a putative adipokinetic hormone/corazonin-related peptide receptor (ACPR) and its cognate ligand were identified from our transcriptome analysis, but unfortunately this GPCR did not bind *C. maenas* ACP, and further investigations into some other incomplete candidate ACPR sequences were abandoned.

CRZR (ORF 1,854 bp, 617 amino acids) and RPCHR (ORF 1,590 bp, 529amino acids) have typical characteristics of rhodopsin-like GPCRs (Prosite). Seven transmembrane domains were predicted (TMHMM) and likewise pfam analysis for CRZR predicted a 7 transmembrane interval between amino acids 115–383, and a disulphide bridge between residues 172 and 250 (Note: ungapped). For RPCHR seven transmembrane domains were predicted between residues 131–395 and a disulphide bridge between cysteines at positions 189–267 (Note: ungapped). For CRZR a divergent DRF motif was found immediately after the predicted 3rd transmembrane domain, for RPCH this corresponded to a conventional DRY motif, and both receptors exhibited the motif NSxxNPxxY in the 7th transmembrane domain; these are typical for rhodopsin GPCRs. Multiple potential N-glycosylation sites (NxS/T) were predicted for the N-terminal extracellular domain (NetNGlyc 1.0 Server). Multiple sequence alignments with other cloned (and deorphanised) CRZRs and RPCH/AKHs show, as expected, high levels of conservation, particularly those areas spanning the 7 transmembrane domains and clear evolutionary relationships (Supplementary Figures 1–3).

### Functional deorphanisation of CRZR and RPCHR

**Figure 2:**
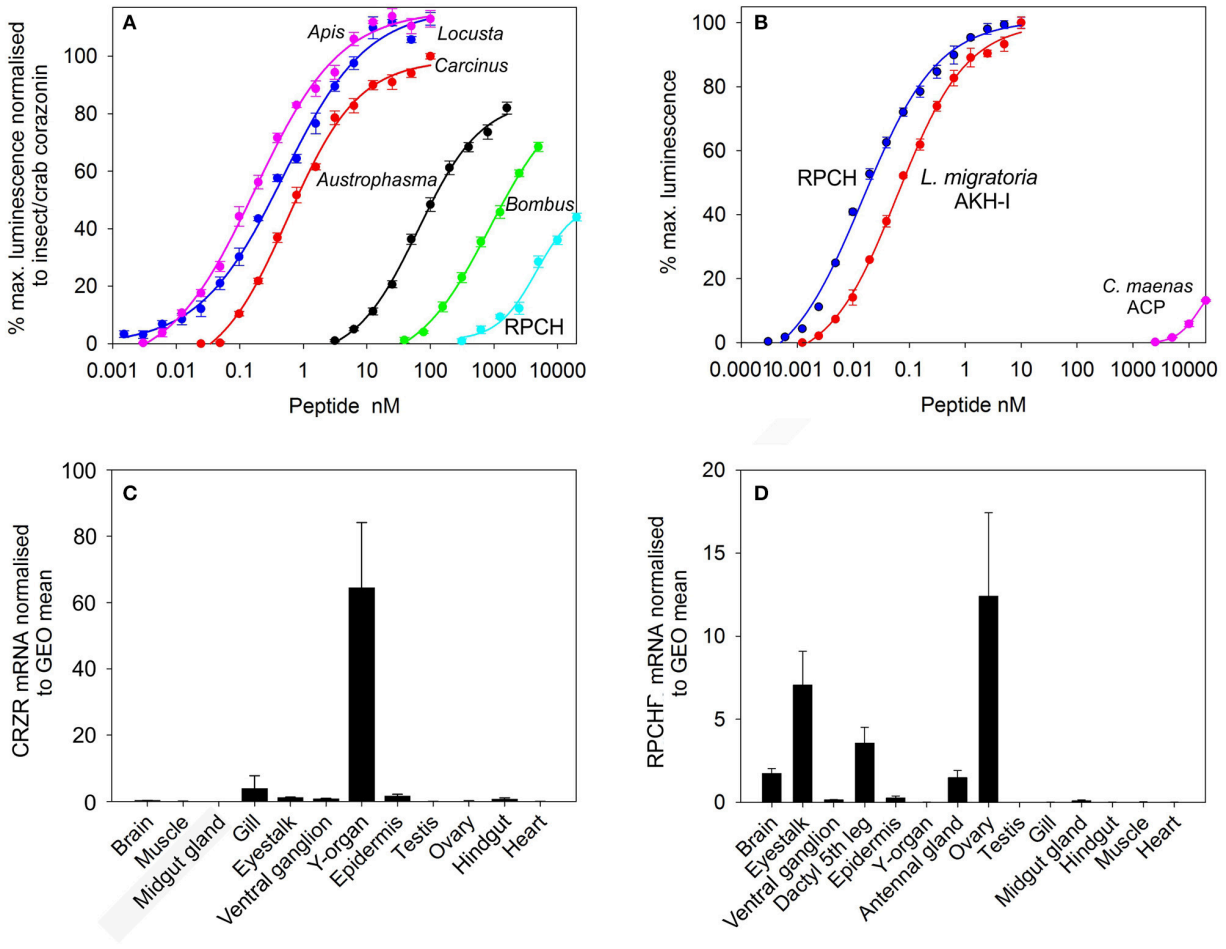
Functional deorphanization of *C. maenas* CRZR and RPCHR. **(A)** Dose response curves of the luminescence response following addition of various CRZs and RPCH to CHO-K1-Aeq cells (without Gα16) transiently expressing CRZR. **(B)** Dose response curves of the luminescence response following addition of RPCH, *Locusta migratoria* AKH-1 and ACP to CHO-K1-Aeq cells (without Gα16) transiently expressing RPCHR. Values are means of *n* = 4 ± 1 SD. **(C)** qRT-PCR showing tissue distribution and abundance of mRNA encoding CRZR. Values are means of *n* = 3 ± 1 SEM, normalized to the geometric (GEO) mean of elongation factor-1 and ubiquitin E3 ligase mRNA. **(D)** qRT-PCR showing tissue distribution and abundance of mRNA encoding RPCHR. Values are means of *n* = 5 ± 1 SEM. Normalisers were GEO means as described above.

Transient expression of cloned CRZR or RPCHR into CHO-K1-Aeq cells either expressing the Gα-16 subunit, or control cells showed equivalent dose response characteristics (Supplementary Figure 4), indicating that these GPCRs signaled via the Gq pathway. For CRZR dose-dependent activation was achieved with an EC_50_ of 0.75 nM (Figure 2A). Curiously, we observed (in several independent experiments) that ligand binding to CRZR with a variety of insect CRZ peptides indicated that those originally isolated from the honey bee, *Apis mellifera* (Thr^4^, His^7^-CRZ) and locust, *Locusta migratoria* (His^7^-CRZ) appeared to give a somewhat higher bioluminescent response at lower doses than the homologous crab/insect ligand (Arg^7^-CRZ). The bioluminescent response of CRZs of the mantophasmoidean, *Austrophasma gansbaaiensis*, (His^4^, Gln^7^-CRZ), (EC_50_ 105 nM), or the hymenopteran, *Bombus soroeensis* (Tyr^3^, Gln^7^, Gln^10^-CRZ) (EC_50_ 1,480 nM) were dramatically reduced (Figure 2A). At extremely high concentrations (0.6–20 μM) some dose-dependent binding of RPCH to CRZR was observed. Receptor binding (expressed as EC_50_ values) together with peptide sequences are summarized on Table 1.

**Table 1:** EC_50_ values calculated from dose response curves of CRZs and RPCH shown in Figure 2A (CRZR), upper panel, and RPCH, AKH-1 and ACP, shown in Figure 2B (RPCHR), lower panel.

*Carcinus maenas* $({\text{R}}^{7}\text{CRZ})\;$	pQTFQYSRGWTN-NH_2_	0.75 nM
*Apis mellifera*	pQTFTYSHGWTN-NH_2_	0.14
*Locusta migratoria*	pQTFQYSHGWTN-NH_2_	0.30
*Austrophasma gansbaaiensis*	pQTFHYSQGWTN-NH_2_	105
*Bombus soroeensis*	pQTYQYSQGWQN-NH_2_	1480
RPCH		>20,000
RPCH	pQLNFSPGW-NH_2_	0.02
*Locusta migratoria* AKH-1	pQLNFTPNWGT-NH_2_	0.07
*Carcinus maenas* ACP	pQITFSRSWVPQ-NH_2_	>>20,000

*Amino acid residues that differ from the prototype peptides (shaded) are shown in red*.

Dose-response curves showing binding to the RPCH receptor are shown on Figure 2B. This receptor bound very low concentrations of RPCH (EC_50_, 20 pM), and also *Lom* AKH-1 (EC_50_, 70 pM). The RPCHR did not bind CRZ (20 μM), but a vanishingly small dose-dependent binding of ACP was observed at micromolar concentrations (Figure 2B, Table 1). Neither receptor was activated by the evolutionarily-related peptide (mammalian) GnRH, even at micromolar concentrations. Control cells transfected with empty vector did not show any luminescent response in the assays.

### Tissue specific expression of CRZR and RPCHR

Expression of CRZR or RPCHR mRNA was examined by qRT-PCR in a variety of crab tissues (Figures 2C,D). For CRZR, low levels of expression were seen in the gill, eyestalk, ventral ganglion, epidermis and hindgut. Levels were undetectable (or at the limit of detection) in the other tissues examined. However, the Y-organ (YO) was notable in consistently showing high levels of expression of this receptor, which exceeded all others combined (Kruskal-Wallace, *P* = 0.002). Similarly, expression of RPCHR was scant or undetectable in several tissues (Figure 2D). However, measurable expression was seen in tissues containing chromatophores, i.e., the dactyl of the fifth walking leg, whilst in other areas of hypodermis, expression was absent. RPCHR was expressed in the nervous system, notably eyestalk neural tissues. Interestingly, ovarian tissues (vitellogenic stages 3-4) showed high but variable levels of RPCHR expression [ANOVA, *F*_(13, 56)_ = 6.016, *P* < 0.001]. *Post hoc* analysis (Tukey's HSD) indicated significant differences in RPCHR expression between ovary and all other tissues, and between the eyestalk and all tissues with the exception of the leg (dactyl) samples.

### Biological activities of CRZ, RPCH, and ACP

#### Chromatophore pigment migration

**Figure 3:**
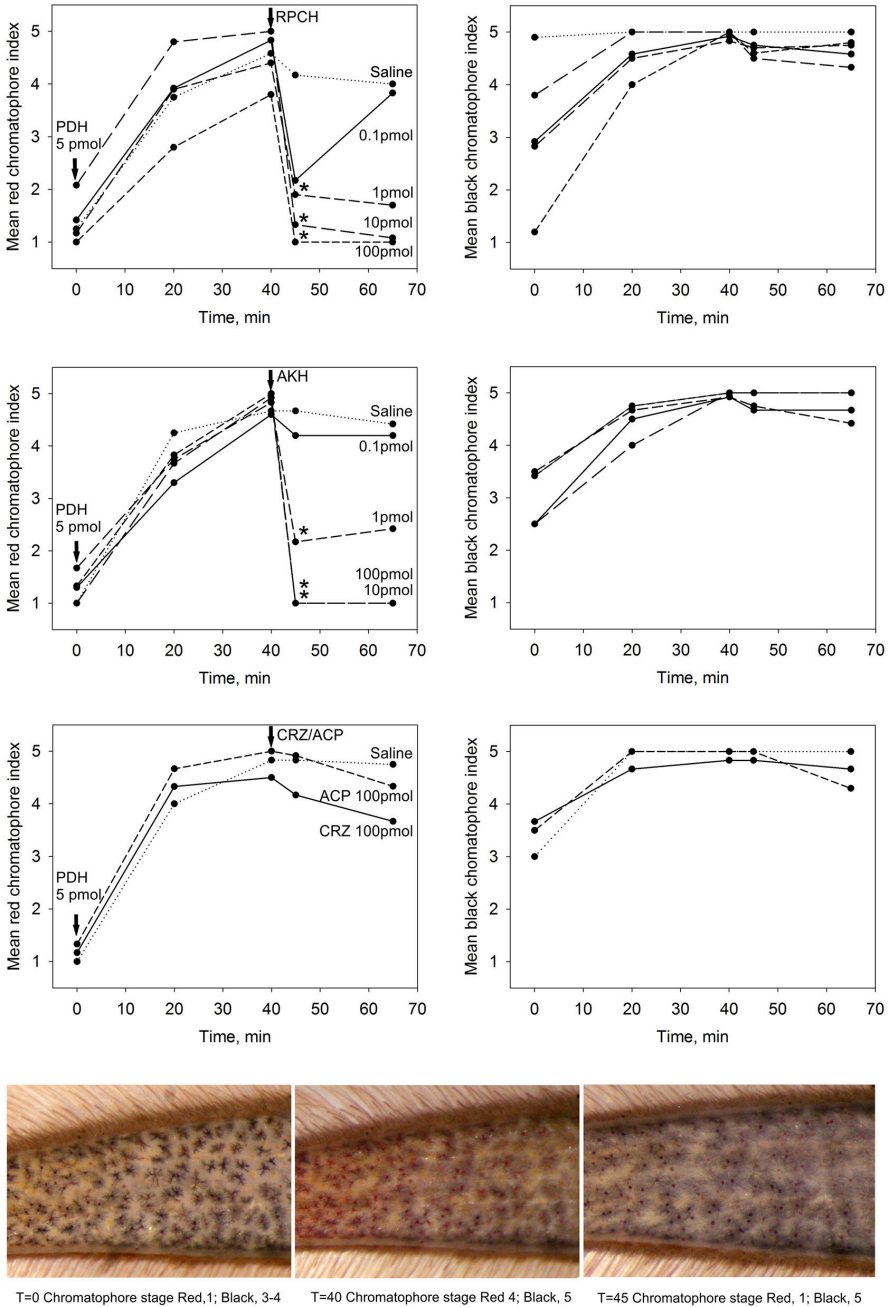
The effect of CRZ, RPCH, AKH, ACP on pigment migration in epidermal chromatophores of the 5th walking leg dactylus. Following dispersion (40 min) of red and black chromatophores by injection of 5 pmol PDH, effectors were injected at 0.1–100 pmol/crab for RPCH or AKH, and 100 pmol/crab for CRZ or ACP. Mean chromatophore indices are indicated. Asterisks indicate significant differences (*p* < 0.01, Wilcoxon-Mann Whitney rank sum test) 5 min after injection of peptide. Lower panel shows a typical response following injection of 1 pmol RPCH.

The biological activity of CRZ, RPCH, and ACP as potential effectors of epidermal pigment migration was investigated by chromatophore bioassay. Following expansion of both red and black chromatophores by injection of 5 pmol PDH over 40 min, pigment concentrating activity of injected peptides was monitored: a summary of the results is shown on Figure 3. As expected, injection of RPCH (100-0.1 pmol) caused a rapid and dramatic (within 5 min.: see images, Figure 3), dose-dependent concentration of pigment in the red (but not the black) chromatophores. Likewise, injection of the non-native, but structurally related insect peptide, *Lom*-AKH-1 was slightly less effective compared to RPCH. Both CRZ and ACP were completely ineffective at causing red or black pigment migration on chromatophores.

#### Ecdysteroid synthesis

**Figure 4:**
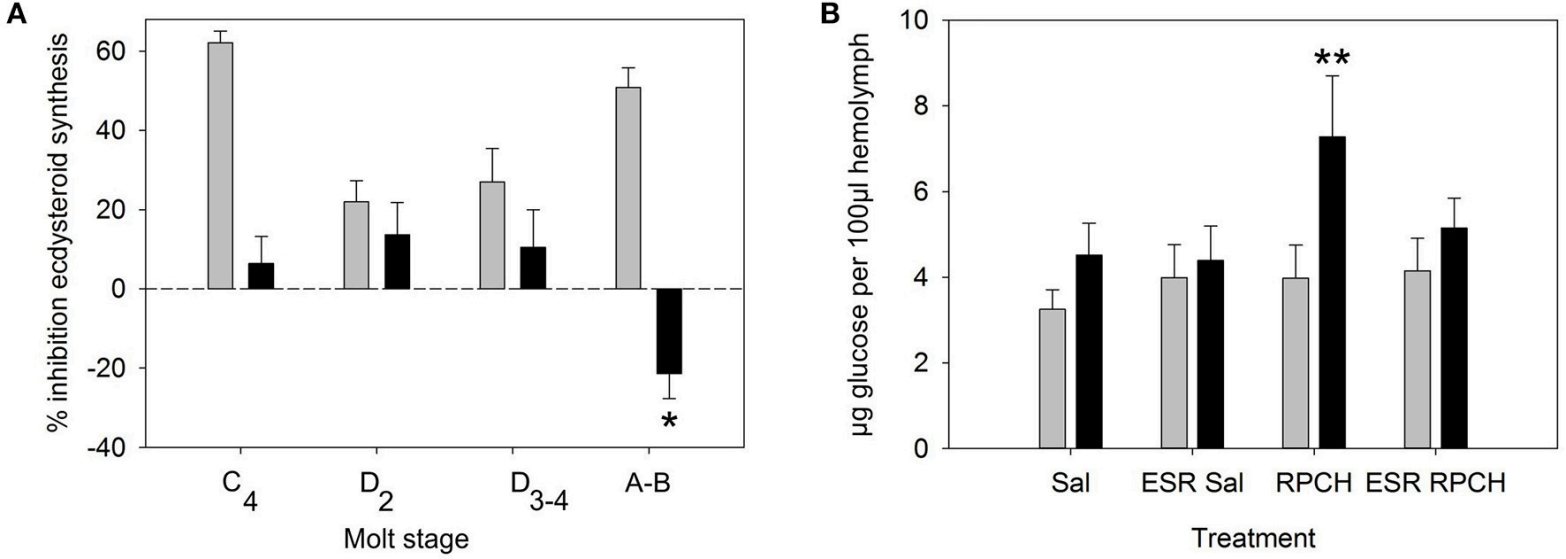
Biological activity of CRZ and RPCH. **(A)** Effect of CRZ on ecdysteroid synthesis by Y-organs *in vitro*. Gray bars = 10 nM MIH, Black bars = 50 nM CRZ, Means ± 1 SEM. *N* = 6 for molt stage C_4_-D_3−4_, 11–13 for **(A,B)**. Asterisk indicates significant stimulation of ecdysteroid synthesis for CRZ at stage A-B (Students matched pair *t*-test, *p* = 0.05). **(B)** The effect of RPCH injection (100 pmol) on hemolymph glucose levels. ESR = eyestalk removal, Sal = saline injection. Gray bars: T = 0, Black bars: *T* = 90 min, Means ± 1 SEM, *n* = 8. Asterisk indicate significant increase in glucose levels (Students matched paired *t*-test *p* < 0.01).

Since the YO expressed relatively high levels of CRZR mRNA compared to other tissues, the possible effects of CRZ on ecdysteroid synthesis was examined over the molt cycle. LC-MS analysis of culture media from YO exposed to 50 nM CRZ (and control media) showed that there were no qualitative changes in the ecdysteroids secreted by the YO when comparing intermolt and premolt samples. In almost every case 25-deoxyecdysone was the principal ecdysteroid secreted, excepting occasional small quantities of 3-dehydroecdysone (Supplementary Table 2). From RIA quantification of ecdysteroids (levels were very similar to those quantified by LC-MS), positive control experiments (MIH, 10 nM) showed inhibition of ecdysteroid synthesis in intermolt, a reduction in inhibition during premolt, and return to high levels of inhibition immediately after molting as expected. However, incubation with high concentrations of CRZ (50 nM) did not inhibit ecdysteroid synthesis in intermolt or during premolt (Figure 4A). The low levels of inhibition observed were statistically non-significant (Student's matched pair *t*-test) Nevertheless, a small, but statistically significant (Student's matched pair *t*-test, *P* = 0.05) stimulation of ecdysteroid synthesis was seen in YO from early postmolt crabs.

#### Glucose and triglyceride levels

Bioassays to measure circulating glucose and triglycerides after injection of CRZ, RPCH and ACP showed that within the dose range investigated (100, 10 pmol per crab), neither CRZ nor ACP exhibited any biological activity (Table 2). However, RPCH injection significantly raised circulating glucose levels within 90 mins of injection (Student's matched pair *t*-test, *P* < 0.01). This effect was indirect: eyestalk removal completely abolished this response (Figure 4B).

**Table 2:** Effect of injected RPCH, CRZ, and ACP on hemolymph triglyceride levels (μg μl^−1^).

**Treatment**	**0 min**	**90 min**
Saline	0.5 ± 0.21	0.7 ± 0.22
RPCH 100 pmol	0.14 ± 0.14	0.86 ± 0.4
CRZ 100 pmol	0.54 ± 0.17	0.39 ± 0.31
ACP 100 pmol	1.47 ± 0.44	1.28 ± 0.34
*RPCH 100 pmol	1.65 ± 0.59	0.78 ± 0.44
*RPCH 10 pmol	0.49 ± 0.27	0.20 ± 0.28

*Values are mean±1 SEM n = 6. All values showed no significant change over 90 min. Asterisk denotes repeat experiment for RPCH*.

#### Myoactivity

**Figure 5:**
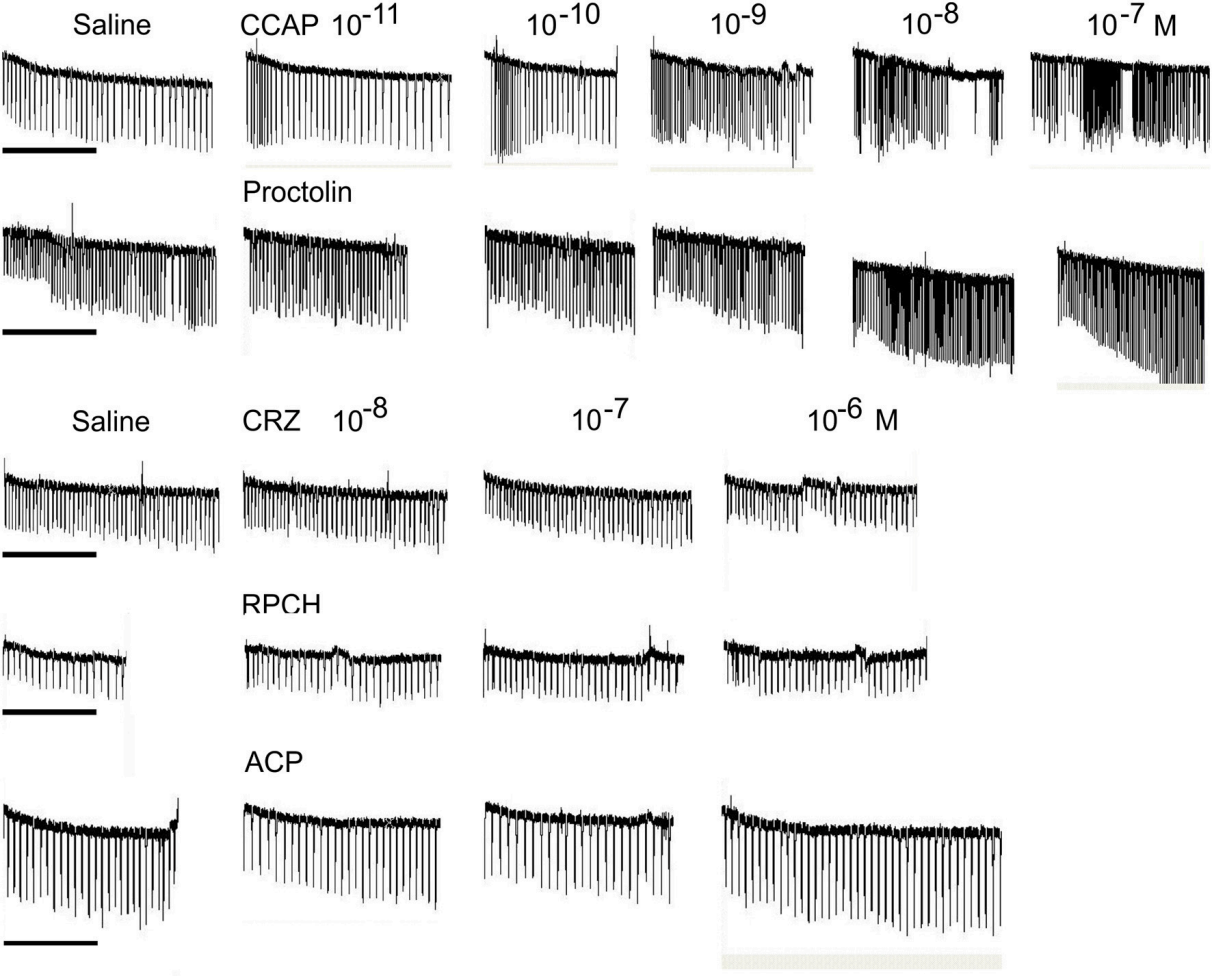
The effect of superfused CRZ, RPCH and ACP upon cardioactivity of semi-isolated heart preparations, compared to those obtained with CCAP or proctolin. Traces show responses from individual crab hearts firstly exposed to saline followed by stepwise 10-fold increases in peptide concentrations. Bars = 1 min.

Superfusive application of CRZ, RPCH, and ACP to semi-isolated hearts showed that these peptides were completely without cardioacceleratory effect at concentrations up to 10^−6^ M (Figure 5). In contrast, application of CCAP showed a threshold response of 10^−10^-10^−11^ M in the most sensitive preparations, and about 10^−9^ M for proctolin, the latter showing a very pronounced inotropic response.

#### Immunohistochemistry and *in situ* hybridization of CRZ, RPCH, ACP transcripts and peptides in the CNS

**Figure 6:**
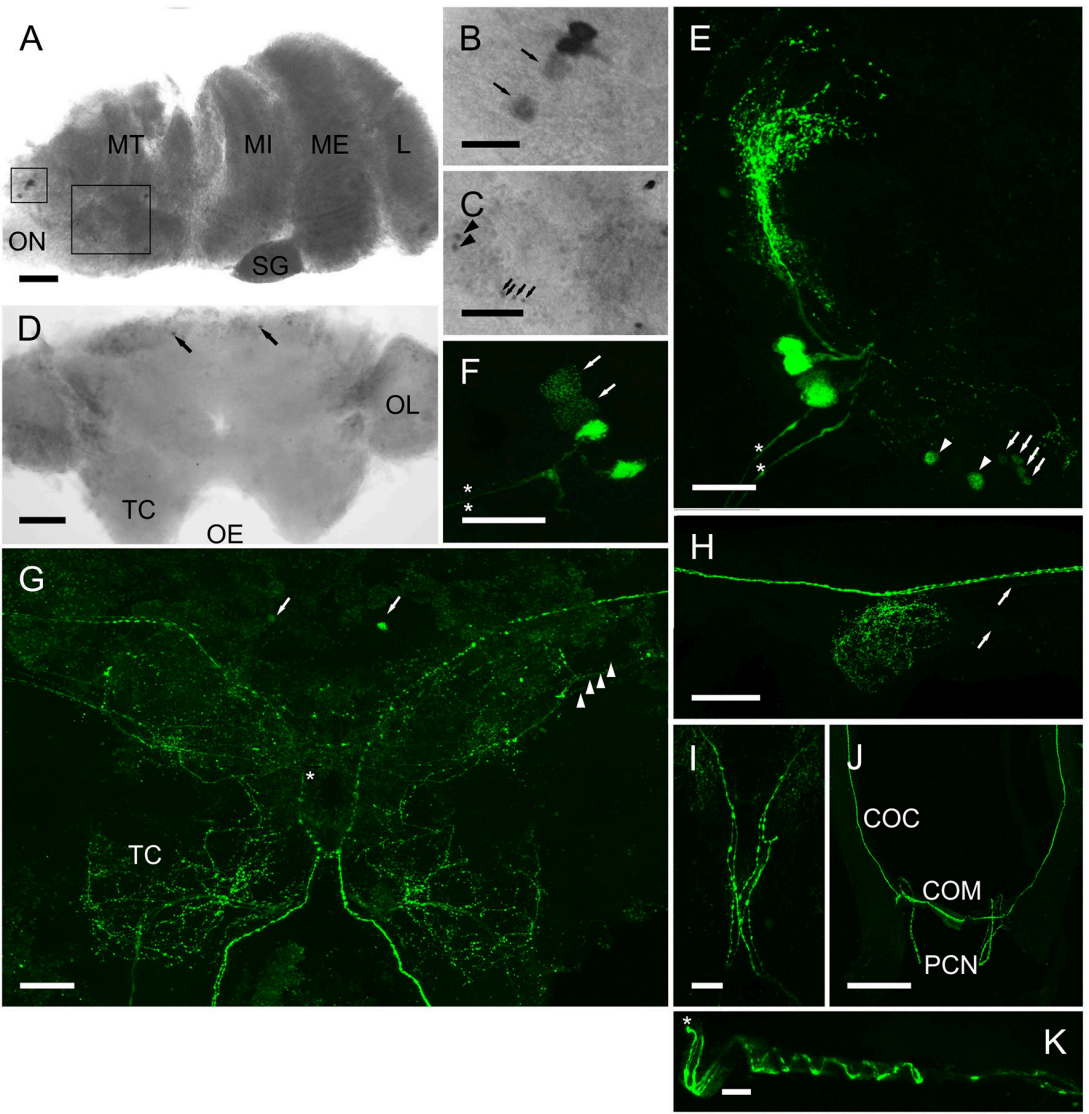
Expression of CRZ mRNA by ISH **(A–D)** and peptide **(E–J)** by ICC and confocal microscopy **(E–J)** (all are maximum intensity projection images) in the CNS of *C. maenas*. **(A)** Overview of CRZ expressing perikarya in the eyestalk. Rectangles highlight CRZ expressing neurones in the medulla terminalis (MT), enlargements are shown on panels **B,C**). **(B)** Small rectangle insert. Two perikarya usually expressed low levels of CRZ mRNA (arrows); this was also seen in protein expression patterns (**F**, arrows). **(C)** Large rectangle insert showing perikarya expressing CRZ mRNA, arrows and arrowheads also refer to equivalent cells in **(E)**. **(D)** Expression of CRZ in the cerebral ganglion by two anterio-dorsal perikarya (arrowheads). **(E)** Four perikarya in the MT, corresponding to those in **(B)**. Arrows and arrowheads correspond to perikarya expressing CRZ mRNA in C. Asterisks highlight two prominent axon bundles in the optic nerve. **(F)** The same cell group shown in **(D)**. In all preparations, two neurones were weakly immunopositive (arrows, see also **B**). Asterisks highlight two prominent axon bundles. **(G)** Overview of CRZ immunopositive structures in the cerebral ganglion. Arrows indicate the two anterio-dorsal perikarya also seen by ISH D). Arrowheads indicate four beaded fibers that project contralaterally (asterisk). The prominent beaded fibers that exit the eyestalk **(E,F)** project axon fibers ipsi- and contra-laterally as shown in **I**). **(H)** Connective ganglion, showing beaded varicosities. Fine descending axons appear to innervate this structure (arrows). **(J)** Circum-oesophageal connectives, commissure and post-commissural nerves. 2-3 descending axons project ipsilaterally across the commissure and exit along the post commissural nerves. **(K)** Post commissural nerve, which is attached to the posterior esophagus (top in image) showing spiral arrangement of axons. Asterisk indicates proximal end of the nerve. COC, circum-oesophageal connective; COM, commissure; MT, medulla terminalis: MI, medulla interna; ME, medulla externa; L, lamina; ON, optic nerve OL, olfactory lobe; PCN post-commissural nerve. Scale bars: **J**, 500 μm; **A,D,G,H**, 200 μm; **E,F,I**, 100 μm; **B,C,K**, 50 μm.

Whole mount *in-situ* hybridization (ISH) using DIG-labeled cRNA probes revealed quite a limited distribution of CRZ neurons in the CNS. The eyestalk (Figures 6A–C) showed expression in a group of 4 perikarya (25–30 μm diam.) adjacent to the optic nerve. In most preparations two of these showed weak hybridization signals, suggesting low levels of transcription. Further groups of small perikarya (10–15 μm) were observed in the dorsal medulla terminalis (Figure 6C). (Note that ISH results in shrinkage of tissues and consequent reduction in estimated cell diameters, whereas the IHC procedure used does not). Confocal microscopy whole mount immunohistochemistry (IHC) using an affinity purified CRZ antiserum clearly demonstrated that the cells identified by ISH contained peptide. The four large perikarya projected processes toward the X-organ (XO), and two prominent axonal bundles exited the eyestalk along the optic nerve (Figures 6E,F). The smaller cells labeled by ISH also labeled. With regard to the four large perikarya, it was notable that in most preparations, two cells contained little immunoreactive material, and labeling of granular structures, presumably Golgi, in these cells suggested a translational nadir. ISH of wholemount cerebral ganglia revealed expression in just two cells (25–30 μm) on the anterio-dorsal margin of the protocerebral midline (Figure 6D). These were also seen in IHC as well as a highly complex arrangement of processes (Figure 6G). The two axon bundles arising from the 4 perikarya in the eyestalk project descending axons- one bundle projects ipsilaterally, whilst the other, contralaterally (Figure 6I). This arrangement was seen in every preparation examined. Fine axons were also observed on the posterior margins of the optic nerve. Whilst these were very difficult to trace, many projected ipsilaterally, terminating in extensive dendritic fields throughout the optic lobes, and in particular the tritocerebrum. Two prominent beaded axon bundles descend along the circum-oesophageal connectives, but although finer branching processes could be observed within the connective ganglia, very fine axons appeared to innervate these structures (Figure 6H, arrows). The prominent beaded axon bundles (2-or 3 could be seen within each connective), project contralaterally directed axons across the commissure; these then enter very fine post-commissural nerves (Figure 6J), which were difficult to trace further; they were too small and fragile to dissect distally. During dissection they appeared to project to the dorsum of the crab on the posterior margin of the esophagus, and in one preparation (Figure 6K), where the oesophageal musculature was not fully dissected, axons in the PCN showed an interesting helical structure indicating that these nerves were probably extendable. These fine, but strongly labeled axons probably terminated in the post-commissural organs (PCO).

**Figure 7:**
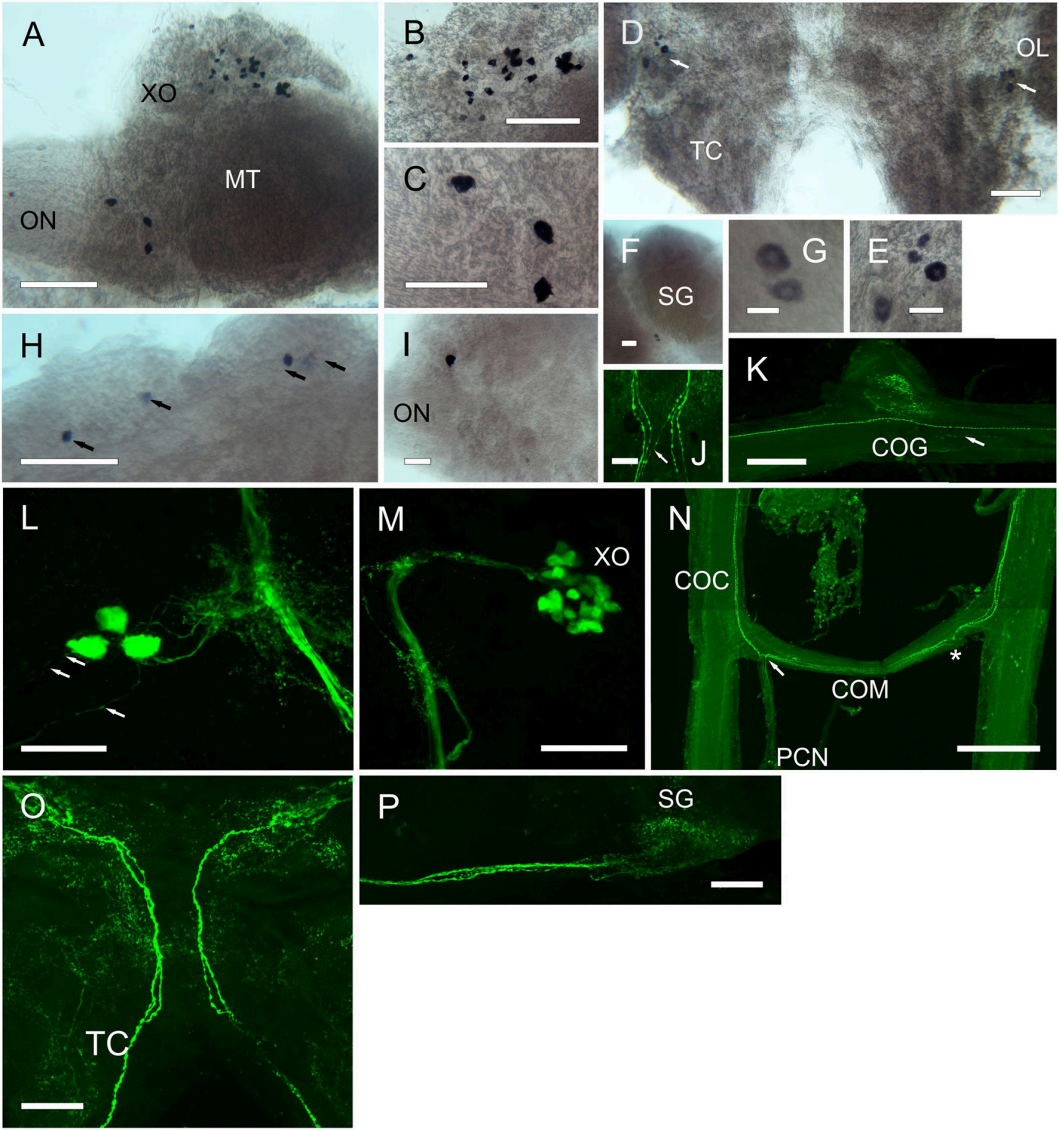
Expression of RPCH and ACP mRNA by ISH **(A–I)** and peptide by ICC and confocal microscopy **(J–P)** (all are maximum intensity projection images) in the CNS of *C. maenas*. **(A)** Overview of RPCH expressing cells in the eyestalk. **(B)** Enlargement of the XO showing a group of around 30 small cells. **(C)** Enlargement of an area of the MT adjacent to the optic nerve showing expression of RPCH in 3cells. **(D)** RPCH expression in the cerebral ganglion showing a group of 4 cells at the junction of the olfactory lobe and tritocerebrum. **(E)** Enlargement of the 4 cells shown in **(D)**. **(F)** Two small cells between the MI and ME, adjacent to the SG. **(G)** Enlargement of the cells in **(F)**. **(H)** ACP mRNA in the cerebral ganglion showing expression by 4 cells at the anterio-dorsal margin. **(I)** A single cell expresses ACP mRNA adjacent to the optic nerve. **(J)** Beaded fibers of descending axons immunopositive for RPCH in the cerebral ganglion. In this image, 2 pairs of fibers project ipsilaterally, and a single fiber, contralaterally. **(K)** RPCH immunoreactive fibers and beaded varicosities in the commissural ganglion. These appear to be innervated by fine descending axons (arrow). **(L)** Three RPCH immunopositive neurons in the medulla terminalis, corresponding to those seen in **(A)**. These perikarya project axons to the sinus gland tract and associated branching dendrites, and project axons to the optic nerve (arrows). **(M)** RPCH neurones in the X-organ. This group of perikarya (seen in A) project a prominent bundle of axons to the sinus gland, as shown in **(P)**. **(N)** Circum-oesophageal connectives, commissure and post commissural nerves showing axons immunopositive to RPCH. 2-3 descending axons project ipsilaterally across the commissure, and exit along the post-commissural nerve. Asterisk indicates position of post-commissural nerve that was removed in this preparation. **(O)** Cerebral ganglion showing two prominent axon bundles exhibiting 2 beaded axons descending to the circum-oesophageal connectives. Many branching dendritic structures are also visible. As shown on Figure 6. Scale bars: **N**, 500 μm; **A,K**, 200 μm; **B–D,H,J,L,M,O,P**, 100 μm; **E,F,I**, 50 μm; **G**, 20 μm.

For RPCH, whole mount ISH (Figure 7) showed two cell groups within the medulla terminalis. Firstly, about 30 small cells (15 μm) within the X-organ, and a group of 3 cells (25–30 μm) at the base of the optic nerve (Figures 7A–C). A pair of small cells (20 μm) was observed between the medulla interna and externa adjacent to the sinus gland (Figures 7F,G). Groups of 4 cells (35, 20 μm) were observed at the junction between the olfactory lobes and tritocerebrum (Figure 7D). For ACP, ISH revealed a group of 4 small cells (25 μm) at the anterio-dorsal midline of the protocerebrum (Figure 7H), and one cell (30 μm) at the base of the optic nerve. However, these were not visible using the ACP antiserum.

Results from whole mount IHC (Figure 7) using an antiserum directed against RPCH confirmed the pattern of mRNA expression seen by ISH: around 30 perikarya (Figure 7M) project axons terminating in the sinus gland (Figure 7P). These neurons show extensive arborizations on the XO-SG tract nearby 3 prominent perikarya (Figure 7L) that correspond with those seen by ISH in the equivalent position in the eyestalk. These three neurons (cell body diameter ca. 30 μm) project processes toward the main XO-SG tract and also direct prominent axons that leave the eyestalk via the optic nerve (Figure 7L). The processes directed toward the XO that were characteristic of CRZ immunoreactivity were not seen using the RPCH antiserum. Likewise, the CRZ antiserum did not label RPCH neurones in the XO, SG tract or SG. The cell bodies showing hybridization signals adjacent to the SG (Figures 7F,G) and in the CG (Figures 7D,E) did not show positive signals by IHC in any preparation. In the cerebral ganglion, axonal fibers enter via the optic nerve in a distinctive pattern of 2 bundles of prominently beaded fibers. These (unlike those immunopositive for CRZ) mostly project ipsilaterally in the tritocerebrum (Figure 7O), but in one preparation, of several examined, a single fiber projected contralaterally (Figure 7J). Within the cerebral ganglion, and particularly within the optic lobes and tritocerebrum, complex branching patterns of immunoreactive processes were observed, but further examination of fine structure was not attempted at this time. The descending axons leaving the CG project along the circum-oesophageal connectives, again showing prominent beaded axons. These do not seem to branch into the commissural ganglia where extensive dendrites were observed- these seem to arise from fine axons (Figure 7K). The prominent descending axons project contralaterally across the circum-oesophageal commissure, and exit via the post-commissural nerves in a pattern similar to that seen for CRZ –IR axonal projections (compare Figures 6J, 7N). Immunopositive structures (or ISH signals) to CRZ, RPCH, or ACP were not observed in any whole mount preparations of ventral ganglia.

## Discussion

In this study we isolated and functionally deorphanised the cDNA sequences encoding CRZR and RPCHR in the crab *Carcinus maenas* and have described receptor distribution, detailed neuroanatomy and physiological roles for both signaling systems, to better understand the evolution and function of these endocrine systems.

It has been proposed that the AKH and ACP receptors originated via a duplication of an ancestral GnRH-like receptor and development of a CRZ receptor (Hansen et al., 2010), with concomitant receptor/ligand coevolution (Cazzamali et al., 2002; Park et al., 2002). This evolutionary scenario is clearly supported in this study; both CRZR and RPCHR show identifiable sequence similarity, not only in the transmembrane domains- as expected since they are rhodopsin- type receptors, but also in the loop and C-terminal regions (Figure 1). The ligands obviously show sequence similarity (Table 1), but neither ligand can effectively activate their heterologous receptors, except for a moderate response at micromolar concentrations. RPCH could activate the CRZR, but conversely CRZ did not activate RPCHR, a feature also noted for the recently deorphanised *Daphnia pulex* (Dappu-) RPCHR (Marco et al., 2017) but curiously, the structurally related peptide ACP showed some receptor binding at micromolar levels- this phenomenon was also noted in that study. In comparison with other CRZRs (functionally deorphanised using the CHO-K1 bioluminescence assay), the Carma-CRZR displays similar dose-response relationships: for the two splice variant *Rhodnius prolixus* CRZRs, EC_50_ values are 2.7 and 1 nM (Hamoudi et al., 2016), *Anopheles gambiae* EC_50_ = 4 nM (Belmont et al., 2006), *Drosophila* EC_50_ = 18 nM (Cazzamali et al., 2002), *Bactrocera dorsalis* EC_50_ = 52 nM (Hou et al., 2017), and for *Manduca sexta* the CRZR binds very low concentrations of CRZ, EC_50_ = 75 pM (Kim et al., 2004). The *C. maenas* CRZR showed rather marked selectivity to CRZ ligands from insects (Figure 2A, Table 1), allowing a simple structure activity analysis. Substitution of R^7^ to H^7^ (*A. mellifera*-CRZ) or Q^3^ to T^3^ (*L. migratoria*-CRZ) did not reduce ligand binding; indeed, we consistently noted in several independent assays that these ligands produced a larger bioluminescent response at lower concentrations than those displayed with the native ligand. Large reductions in binding were noted for Mantophasmoidea (*A.gansbaaiensis*-CRZ) and *B. soroeensis*-CRZ. Since for the latter, a conservative substitution F^3^-Y^3^ would seem to be trivial, it seems likely that the Q substitutions at the positions 7 and 10 would point to the importance of the C-terminus in receptor binding.

The RPCH receptor showed rather higher sensitivity binding to its cognate ligand (EC_50_ = 20 pM) or related peptide Lom AKH-1 (75 pM) than CRZR to its cognate receptor (0.75 nM). The high sensitivity binding of RPCHR was also noted for Dappu-RPCHR (64 pM) and a similarly modest reduction of binding to insect AKHs (Marco et al., 2017). In comparison the AKHR of insects seems to bind homologous ligands at higher concentrations including *Drosophila* EC_50_ = 0.8 nM (Staubli et al., 2002), *Bombyx mori* EC_50_ = 6.4 nM AKH1, 11.7 nM AKH2 (Zhu et al., 2009). This was notable for the AKHR of *Glossina mortisans* where EC_50_ values were in the high nanomolar to micromolar range (Caers et al., 2016). For the *D. pulex* RPCHR, stepwise substitution of alanine at any of residues 1-4 or 8 were important in receptor binding, and in particular substitution of F_4_ with A_4_ resulted in a dramatic reduction in binding (Marco et al., 2017).

In order to further define possible roles of CRZ and RPCH we were interested in quantifying receptor expression in tissues. The first inkling of an unusual distribution of CRZR, and indeed its discovery in *C. maenas*, came from *in silico* transcriptomic studies where we subtracted identified GPCR transcripts from Y-organ (YO) and epidermal transcriptomes. This process revealed a GnRHR-type transcript, uniquely expressed in the YO, subsequently deorphanised here as that of a CRZR. Subsequent comprehensive qPCR experiments showed that apart from the YO few other tissues express significant levels of CRZR mRNA. These results were in marked contrast to that detailed for *Rhodnius prolixus*, where CRZR expression was promiscuous, and in particular, high levels of expression were seen in the CNS, dorsal vessel, abdominal dorsal epidermis, and notably, the prothoracic gland (Hamoudi et al., 2016). These results were intriguing and suggestive of rather different functions of corazonin in insects and crustaceans.

qPCR to show the abundance of RPCHR mRNA revealed, as expected, that high levels of the receptor were expressed in the eyestalk neural tissue, which is in accord with the well-known actions of the peptide upon retinal pigment migration and sensitivity (Gaus and Stieve, 1992; Garfias et al., 1995). The very low level of expression observed in the epidermis was initially surprising, but in many areas of adult crab epidermis, which is overlaid by a thick calcified cuticle, chromatophores are scant or absent. However, when samples were taken from the tip (dactylus) of the 5th walking leg of juvenile crabs, where calcification is minimal, the cuticle thin and transparent, and which contains an abundance of chromatophores RPCHR expression was easily detectable. Apart from low level expression of receptor in the antennal gland, we observed expression of RPCHR in the mature (stage 3-4) ovary. Whilst this tissue distribution might only represent expression of maternal mRNA, as we have observed for several neuropeptides in early embryonic development (Chung and Webster, 2004), a recent report indicates that RPCH is expressed in the maturing ovary and may be involved in oocyte maturation in the crab *Scylla paramamosain* (Zeng et al., 2016). However, in that study the action of RPCH seems to be indirect in that other (unidentified) factors seem to be involved together with RPCH. Nevertheless, given our findings that RPCHR is expressed in the maturing ovary, a novel role for RPCH in reproduction seems possible.

Given the distribution of mRNAs encoding CRZR and RPCHR, we accordingly designed bioassays to attempt to discover roles for their cognate ligands. Corazonin was completely without cardioactivity in semi-isolated heart preparations, as were RPCH and ACP, even at micromolar concentrations (Figure 5). This was surprising, given the well-known action of corazonin on increasing heart rate in some insects (Veenstra, 1989; Patel et al., 2014), notwithstanding the report that knockdown of corazonin and CRZR receptor in *Anopheles gambiae* had no effect on heart rate (Hillyer et al., 2012). It is also relevant to note that heart tissue did not express CRZR at detectable levels by qPCR. Corazonin (and ACP) displayed no activity in chromatophore bioassays, whereas as expected, RPCH (and AKH) were exquisitely potent in concentrating red chromatophores as shown on Figure 5. (N.B. None of these peptides caused melanophore pigment concentration). Bioassays to measure lipid and carbohydrate (glucose) mobilization showed that CRZ, RPCH and ACP were completely without effect on hemolymph diacylglycerol levels (Table 1). However, RPCH injection causes hyperglycemia in intact, but not eyestalkless crabs (Figure 4B). Thus, it is possible to speculate that a novel role for this neuropeptide could be in releasing crustacean hyperglycemic hormone (CHH), whose action in eliciting hyperglycemia is perhaps the best known of any crustacean neurohormone (review Webster et al., 2012).

Corazonin did not affect ecdysteroid synthesis by Y-organs *in vitro* at any stage of the molt cycle, excepting a small stimulation in postmolt. The positive control experiments (MIH 10 nM) were designed to maximally repress ecdysteroid synthesis during intermolt, as detailed previously (Chung and Webster, 2003), clearly showed maximum repression of ecdysteroid synthesis in intermolt, which declined to low values during premolt, and subsequently, YO regained competence to respond to MIH in early postmolt. The high levels of CRZ used, that we consider to be supra-maximal in a physiologically relevant context were without effect in repressing ecdysteroid synthesis in intermolt or premolt. This is in accord with results for *R. prolixus*, where RNAi of CRZR did not affect ecdysis timing (Hamoudi et al., 2016). Whilst it might be premature to speculate at this point, the almost exclusive expression of CRZR on a tissue in which the only established function is concerned with ecdysteroid synthesis, is certainly worthy of this. A key role of corazonin in larval moth (*M. sexta*) ecdysis is in stimulating release of PETH and ETH from Inka cells, thus triggering the cascade of neuropeptide release in ecdysis (see Žitnan and Adams, 2012 for a recent review). As yet, it is not known whether the YO has any neuroendocrine function; its ultrastructure is certainly not indicative of protein synthesis in crabs including *C. maenas* (Bucholz and Adelung, 1980), and closely associated neurohemal structures analogous to the lateral cephalic nerve plexus of isopods (Besse and Legrand, 1964) are not present in decapods. Nevertheless, it would certainly be worthwhile to revisit this issue, using contemporary technologies. A further possibility was that we had inadvertently identified the MIH receptor, but which fortuitously also bound CRZ. We can discard this hypothesis, not only because CRZ has no activity on inhibition of ecdysteroid synthesis by YO *in vitro*, but also because MIH did not activate the cloned CRZR in the luminescence assay, even at micromolar concentrations.

The anatomy of the CRZ, RPCH, and ACP neuronal systems, investigated by ISH and IHC indicated that these were restricted to the eyestalk ganglia, brain and oesophageal nerves; no neurons were identified in the ventral ganglia. For RPCH, neuronal architecture was broadly similar to that reported for *C. maenas* (Mangerich et al., 1986) but immunopositive neurons were not observed the ventral ganglia, in contrast to the situation in many insects where segmentally iterated neurons and complex branching dendrite arrangements are seen throughout the sub-oesophageal, thoracic and abdominal ganglia, apart from the protocerebral neurons that direct descending axons (ipsilaterally) to the release sites in the corpora cardiaca (Roller et al., 2003). Of particular interest was the anatomy of CRZ immunopositive neurons since these have not been reported in crustaceans to date. Given the similarity in sequence between RPCH, CRZ, and ACP, how confident are we that these neuronal systems are anatomically distinct?

ISH showed that apart from the population of around 30 RPCH cells in the XO, a small population of perikarya expressing RPCH (3 cells) CRZ (4 cells) and ACP (1 cell) were localized dorsally, near the exit point of the optic nerve. IHC for RPCH and CRZ corroborated this finding. Furthermore, since the CRZ antiserum did not label the RPCH neurons, as evidenced by a complete absence of labeled structures in the SG and XO corresponding to RPCH, and the absence of labeling of unique CRZ immunopositive structures, such as the processes extending to the XO and small ventral cells (Figure 6E), indicate that labeling was highly specific. The ACP antiserum proved not to have a high titre, and only labeled RPCH neurons, rather faintly (results not shown), so the neuronal morphology of cells producing this peptide could not be examined further. The arrangement of descending neurons through the cerebral ganglia was very similar for RPCH and CRZ, excepting that the crossing over arrangement of ipsilateral and contralateral axons in the tritocerebrum which was different for both peptides (Figures 6B, 7O). However, the arrangement of axonal projections within the circum-oesophageal connectives, commissure and post-commissural nerves was strikingly similar. Since the anatomy of these neurons from the cell bodies to post-commissural nerves was so similar, we cannot yet say for certain whether these cell groups produce both peptides. Several attempts at direct labeling of the antisera with Alexa 594 Fluor (Invitrogen) for double labeling proved unsuccessful for both antisera; labeling dramatically reduced their antigenicity (despite the effectiveness of this technique in labeling several other antisera). However, the most significant finding of these neuroanatomical studies concerns the release sites of CRZ (and RPCH). The post-commissural nerves have long been known to project dorsally to the so-called post-commissural organs (PCO) (Knowles, 1953; Maynard, 1961a,b), yet the peptides secreted from the PCO have been poorly characterized, excepting a mention that they contain pigmentary effector hormones (presumably RPCH) (Fingerman, 1966). Thus, this neuroanatomical study clearly suggests that both RPCH and CRZ are secretable neurohormones that are probably released from the post-commissural organs. Further studies on the fine structure of this long-known but poorly understood neurohemal tissue are now timely.

This study has shown that deorphanisation of ligand/receptor pairs, coupled with gene and peptide expression studies has the potential to be an effective strategy in functional neuroendocrinology. For corazonin, it now seems clear that the original name- based solely upon a rather modest cardioacceleratory effect in very few species of insects needs to be reconsidered. The accumulating body of evidence points to notably diverse roles for this peptide, and indeed to other members of the CRZ/AKH/ACP family and the tantalizing glimpse of a role for CRZ in control of ecdysis suggested from this, and insect studies, should stimulate further research. Knockdown strategies should reveal gene regulatory networks, and by interrogating tissue and molt-stage specific transcriptomes, it is now possible and timely to dramatically accelerate functional studies of arthropod neuropeptides.

## Author contributions

SW and DW designed the research. JA, AO, SW, DW, NA, RD, and RL performed the research. SW, JA, AO, and DW wrote the paper.

### Conflict of interest statement

The authors declare that the research was conducted in the absence of any commercial or financial relationships that could be construed as a potential conflict of interest.

## Supplementary material

Supplementary Figure 1 Amino acid sequence alignments of Carma-CRZR (MF974386), compared with other cloned and functionally deorphanised receptors. **CRZR**: *Drosophila melanogaster* (Cazzamali et al., 2002) (CG10698); *Manduca sexta* (Kim et al., 2004) (AY369029); *Anopheles gambiae* (Belmont et al., 2006) (AY301275); −α,β *Rhodnius prolixus* (Hamoudi et al., 2016) (KU052880, KU052881); *Bactrocera dorsalis* (Hou et al., 2017) (KX831394).

Supplementary Figure 2 Amino acid sequence alignments of Carma-RPCH (MF974387), compared to other cloned and functionally deorphanised receptors. **RPCHR/AKHR**: *Drosphila melanogaster, Bombyx mori* (Staubli et al., 2002) (AF077299, AF403542); *Glossina mortisans*–A, −B (Caers et al., 2016) (GMOY008368 (VectorBaseID), HQ640948); *Daphnia pulex* (Marco et al., 2017) (KY426816).

Supplementary Figure 3 A neighbor-joining tree of the deorphanised CRZ/RPCH/AKH receptors as referred to in Figure 1. Analysis was performed using Geneious Tree Builder v 9.1.8. Bootstrap supported values are based on 100 replicates using the Jukes-Cantor genetic distance model. The AKH/corazonin-related peptide receptor (ACPR) of *Anopheles gambiae* (EU138885; Hansen et al., 2010) was used as an outgroup to root the tree.

Supplementary Figure 4 Dose response curves of the luminescence response for CHO-KI-Aeq cells with Gα16 subunit (blue) or control cells (red), transiently expressing CRZR **(left)** or RPCHR **(right)**, and exposed to their homologous ligands Values are means ± 1 SD of quadruplicate measurements.

Supplementary Table 1 Primer and probe sequences for quantitative Taqman PCR and *in situ* hybridization. Each Taqman assay includes a forward and reverse primer (F′ and R′) and a hydrolysis probe labeled at the 5′ end with a fluorescent dye and a minor groove binding 3′ end. “Standard synthesis” refers to primer pairs used in the generation of PCR templates for in vitro transcription of qPCR standard curves. The T7 phage promoter site (T7) used had the sequence 5′-TAATACGACTCACTATAGGG-3′.

Supplementary Table 2 LC-MS analysis of ecdysteroids secreted by YO pairs *in vitro*. Blue numbers indicate samples from premolt (D2), red numbers, intermolt YO pairs. YO exposed to 50nM CRZ are denoted by A. Asterisks indicate probable loss of sample. Loq = limit of quantification by multiple reaction monitoring (MRM). Detection limits were: ecdysone, 250ng/ml, (none detected in samples), 3-dehydroecdysone (3dE), 25-deoxyecdysone 10ng/ml. The values obtained are broadly similar to those measured by RIA for molt staged YO in this (and our previous) studies. Enzyme immunoassays (using a variety of different antisera and peroxidase labelled competitor) give erroneous measurements in YO bioassays due to the variable presence of endogenous peroxidases.

## Abbreviations

ACP: adipokinetic hormone/corazonin-related peptide
ACPR: adipokinetic hormone/corazonin-related peptide receptor
AKH: adipokinetic hormone
BSA: bovine serum albumin
CCAP: crustacean cardioactive peptide
CG: cerebral ganglion
CHH: crustacean hyperglycemic hormone
CHO: Chinese hamster ovary
CNS: central nervous system
CRZ: corazonin
CRZR: corazonin receptor
DBT: doubletime
DIG: digoxygenin
DMEM: Dulbecco's Modified Eagle Medium
ETH: ecdysis triggering hormone
GnRH: gonadotropin releasing hormone
GPCR: G protein-coupled receptor
IHC: immunohistochemistry
ISH: in situ hybridization
LC-MS: liquid chromatography - mass spectrometry
MIH: molt-inhibiting hormone
ORF: open reading frame
PCN: pericardial nerve
PCO: post-commissural organ
PDH: pigment dispersing hormone
PER: period
PETH: pre-ecdysis triggering hormone
PFA: paraformaldehyde
RIA: radioimmunoassay
RPCH: red pigment concentrating hormone
RPCHR: red pigment concentrating hormone receptor
SG: sinus gland
XO: X-organ
YO: Y-organ.
