# Supplementary figures and images for: Functional Characterization and Signaling Systems of Corazonin and Red Pigment Concentrating Hormone in the Green Shore Crab, Carcinus maenas
Source: Front Neurosci. 2018 Jan 15;11:752. doi: 10.3389/fnins.2017.00752 (PMC5775280; doi:10.3389/fnins.2017.00752)

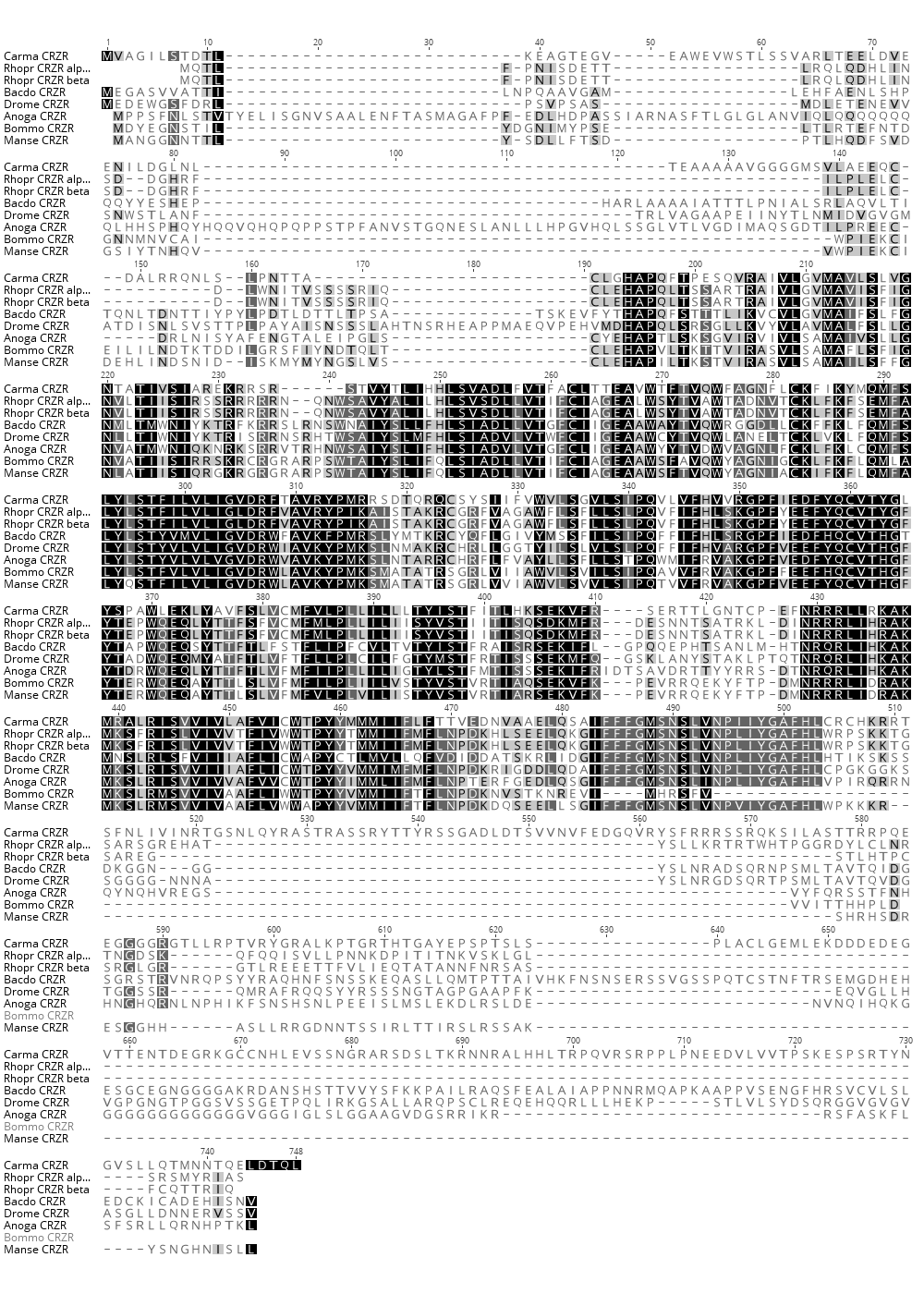

Supplement: Supplementary Figure 1 — Amino acid sequence alignments of Carma-CRZR (MF974386), compared with other cloned and functionally deorphanised receptors. CRZR: Drosophila melanogaster (Cazzamali et al., 2002) (CG10698); Manduca sexta (Kim et al., 2004) (AY369029); Anopheles gambiae (Belmont et al., 2006) (AY301275); −α,β Rhodnius prolixus (Hamoudi et al., 2016) (KU052880, KU052881); Bactrocera dorsalis (Hou et al., 2017) (KX831394). [file Image1.PNG]

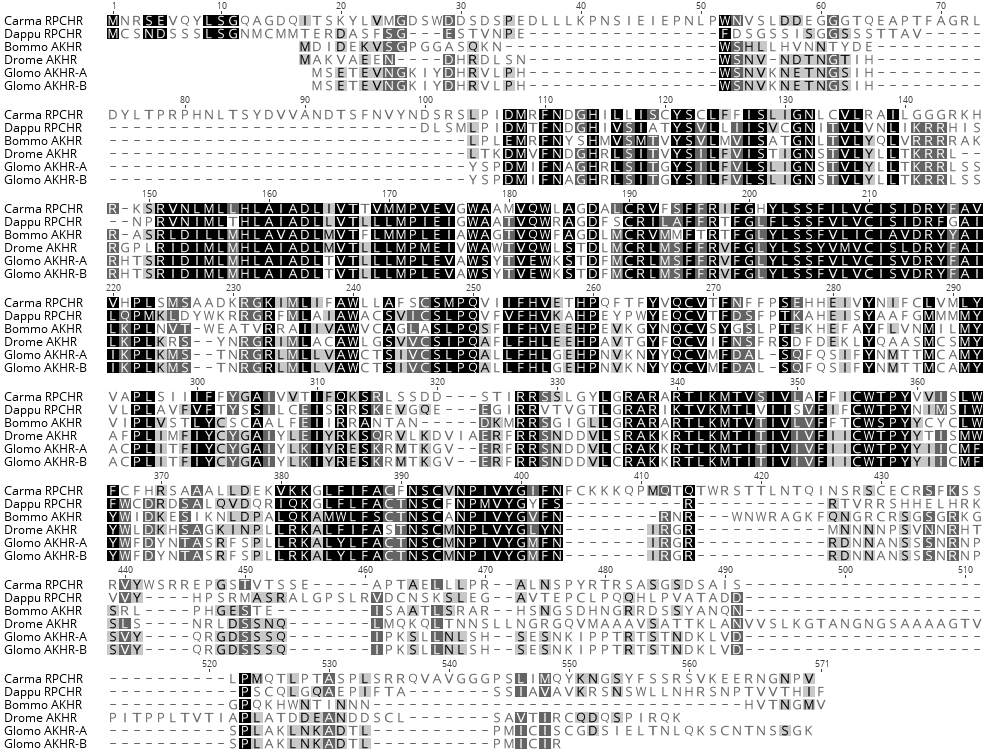

Supplement: Supplementary Figure 2 — Amino acid sequence alignments of Carma-RPCH (MF974387), compared to other cloned and functionally deorphanised receptors. RPCHR/AKHR: Drosphila melanogaster, Bombyx mori (Staubli et al., 2002) (AF077299, AF403542); Glossina mortisans–A, −B (Caers et al., 2016) (GMOY008368 (VectorBaseID), HQ640948); Daphnia pulex (Marco et al., 2017) (KY426816). [file Image2.PNG]

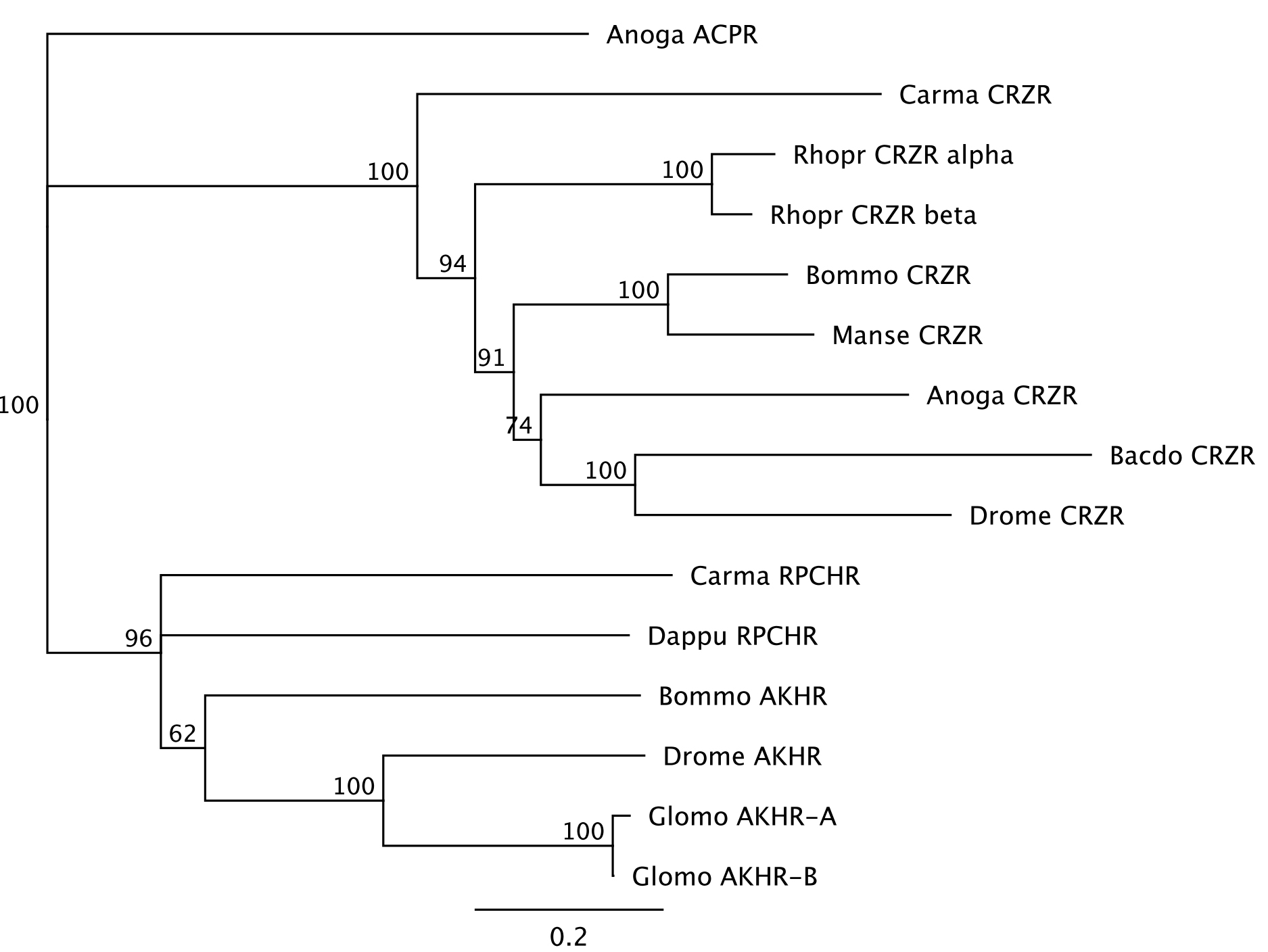

Supplement: Supplementary Figure 3 — A neighbor-joining tree of the deorphanised CRZ/RPCH/AKH receptors as referred to in Figure 1. Analysis was performed using Geneious Tree Builder v 9.1.8. Bootstrap supported values are based on 100 replicates using the Jukes-Cantor genetic distance model. The AKH/corazonin-related peptide receptor (ACPR) of Anopheles gambiae (EU138885; Hansen et al., 2010) was used as an outgroup to root the tree. [file Image3.JPEG]

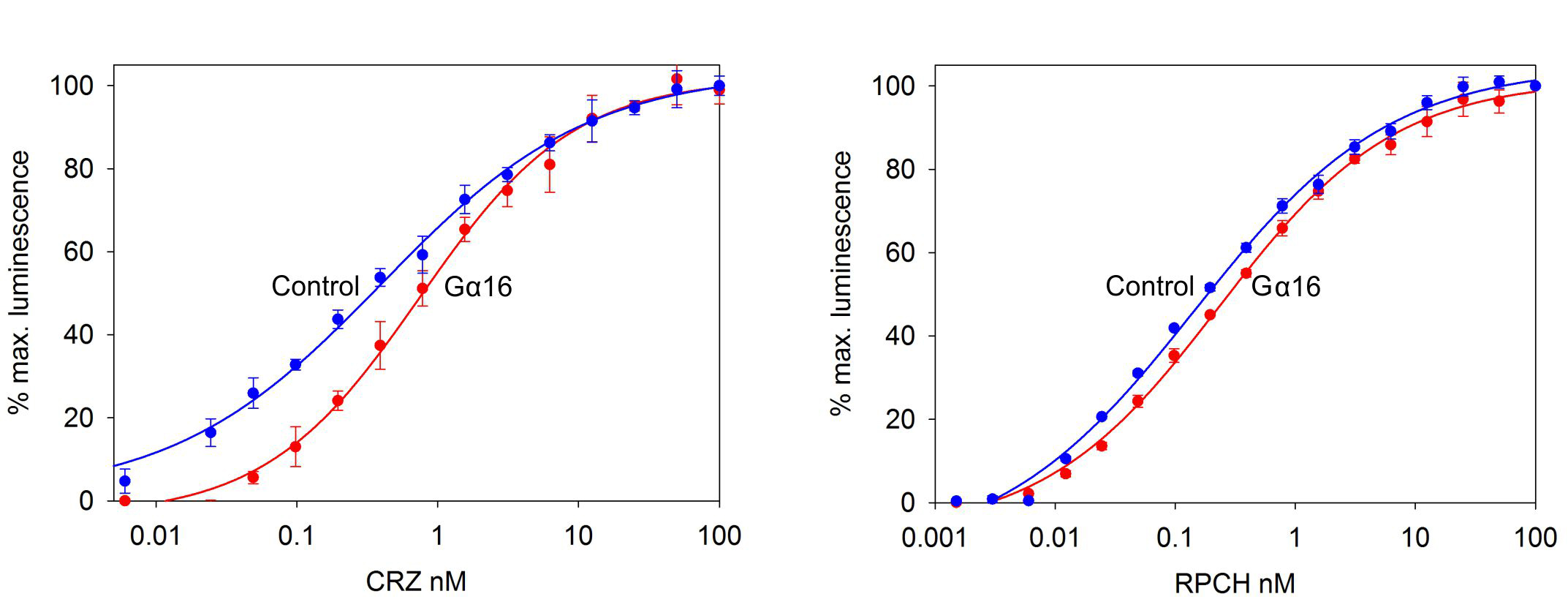

Supplement: Supplementary Figure 4 — Dose response curves of the luminescence response for CHO-KI-Aeq cells with Gα16 subunit (blue) or control cells (red), transiently expressing CRZR (left) or RPCHR (right), and exposed to their homologous ligands Values are means ± 1 SD of quadruplicate measurements. [file Image4.JPEG]
