# Supplementary material for: Functional Characterization and Signaling Systems of Corazonin and Red Pigment Concentrating Hormone in the Green Shore Crab, Carcinus maenas
Source: Front Neurosci. 2018 Jan 15;11:752. doi: 10.3389/fnins.2017.00752 (PMC5775280; doi:10.3389/fnins.2017.00752)
Supplement: Supplementary Table 1 — Primer and probe sequences for quantitative Taqman PCR and in situ hybridization. Each Taqman assay includes a forward and reverse primer (F′ and R′) and a hydrolysis probe labeled at the 5′ end with a fluorescent dye and a minor groove binding 3′ end. “Standard synthesis” refers to primer pairs used in the generation of PCR templates for in vitro transcription of qPCR standard curves. The T7 phage promoter site (T7) used had the sequence 5′-TAATACGACTCACTATAGGG-3′. [file Table1.DOCX]

**SUPPLEMENTARY TABLES Table 1. Primer and probe sequences for quantitative Taqman PCR and in situ hybridization**. Each Taqman assay includes a forward and reverse primer (F’ and R’) and a hydrolysis probe labeled at the 5’ end with a fluorescent dye and a minor groove binding 3’ end. ‘Standard synthesis’ refers to primer pairs used in the generation of PCR templates for in vitro transcription of qPCR standard curves. The T7 phage promoter site (T7) used had the sequence 5’-TAATACGACTCACTATAGGG-3’.

| Method | Oligonucleotide name | Sequence (5’-3’) |
| --- | --- | --- |
|  |  |  |
| *CRZR* Taqman qPCR assay | CamCRZR Taqman probe | NED-TGCGAGCCCTCCG-MGB |
|  | CamCRZR Taqman F’ | AGGCTGCTGAGGAAGGCTAAG |
|  | CamCRZR Taqman R’ | CCAACACGATCACCACAGAGA |
| *RPCHR* Taqman qPCR assay | CamRPCHR Taqman probe | FAM-CATGCCACAGGTAAT-MGB |
|  | CamRPCHR Taqman F’ | ATGCGTCTCCACGTGAAAGAT |
|  | CamRPCHR Taqman R’ | GGCTCTTGGCGTTTAGCTGTT |
| *UbcE2L3* Taqman qPCR assay | CamUbcE2 Taqman probe | FAM-ACCCGAGAACCCACC-MGB |
|  | CamUbcE2 Taqman F’ | TCACCTGGCAGGGACTCATT |
|  | CamUbcE2 Taqman R’ | CCTGAACGCTCCCTTGTTGT |
| *Elf1a* Taqman qPCR assay | CamElf1a Taqman probe | VIC-CTCTCTTTGACGCTCTGG-MGB |
|  | CamELF1a Taqman F’ | GAGCGGCAGCTATGAGTTCAT |
|  | CamElf1a Taqman R’ | TGGATGGAGGCTCAATGTTG |
|  |  |  |
| *CRZR* standard synthesis | CamCRZR STD F’ | AGGCTGCTGAGGAAGGCTAAG |
|  | CamCRZR STD T7 R’ | T7-AGGAAGTCCTTCGCTTGTGA |
| *RPCHR* standard synthesis | CamRPCHR STD T7 F’ | T7-CGGCAATCTGTGTGTACTGC |
|  | CamRPCHR STD R | TGGAGAGAGGAGCCACGTAT |
| *Elf1a* standard synthesis | CamElf1a STD T7 F’ | T7-CCAAGATCGAGCGTAAGAGC |
|  | CamElf1a STD R’ | TTTCACAGCTCAGGTGATCG |
| *UbcE2L3* standard synthesis | Cam UbcE2 STD T7 F’ | T7-ACATTCGAAGGTCTGGCATC |
|  | Cam UbcE2 STD R’ | CCAAAGACCGCAAGAAGTTC |
|  |  |  |
| *CRZ in situ* hybridization probe synthesis | CamCRZ DIG FT7’ | T7-CTCCGTGCCAGGTTGTCTAT |
|  | CamCRZ DIG RT7’ | T7-GTGTGGGAAGCGTTGTTTCT |
|  | CamCRZ DIG F’ | CTCCGTGCCAGGTTGTCTAT |
|  | CamCRZ DIG R’ | GTGTGGGAAGCGTTGTTTCT |
| *RPCH in situ* hybridization probe synthesis | RPCH 15 F | CTTCAGGTTCTCAACTGTCTC |
|  | RPCH 15 F T7 | T7-CTTCAGGTTCTCAACTGTCTC |
|  | RPCH 460 Rev | GAGGAGAACATCTATGGGGT |
|  | RPCH 460 Rev T7 | T7-GAGGAGAACATCTATGGGGT |
| *ACP in situ* hybridization probe synthesis | ACP 61 F | TCTTGTGTGTTGATGGACAG |
|  | ACP 434 Rev | GTTTGTTGTTAGTGAAGGTGTT |
|  | ACP 61 F T7 | T7-TCTTGTGTGTTGATGGACAG |
|  | ACP 434 Rev T7 | T7-GTTTGTTGTTAGTGAAGGTGTT |
|  |  |  |
| *CRZR* directional cloning | CRZR 150 F | CACCCTCGGCATGGTGGC |
|  | CRZR 1990 R | CTCCTCAAAGCTGCGTATCCAACTC |
| *RPCHR* directional cloning | TR77839 927 F (RPCHR) | CACCATGAACCGTTCTGAAGTT |
|  | TR77839 2543 R (RPCHR) | CCTTACACTACATTGACTTGCTGTTA |
