# Supplementary material for: Functional Characterization and Signaling Systems of Corazonin and Red Pigment Concentrating Hormone in the Green Shore Crab, Carcinus maenas
Source: Front Neurosci. 2018 Jan 15;11:752. doi: 10.3389/fnins.2017.00752 (PMC5775280; doi:10.3389/fnins.2017.00752)
Supplement: Supplementary Table 2 — LC-MS analysis of ecdysteroids secreted by YO pairs in vitro. Blue numbers indicate samples from premolt (D2), red numbers, intermolt YO pairs. YO exposed to 50nM CRZ are denoted by A. Asterisks indicate probable loss of sample. Loq = limit of quantification by multiple reaction monitoring (MRM). Detection limits were: ecdysone, 250ng/ml, (none detected in samples), 3-dehydroecdysone (3dE), 25-deoxyecdysone 10ng/ml. The values obtained are broadly similar to those measured by RIA for molt staged YO in this (and our previous) studies. Enzyme immunoassays (using a variety of different antisera and peroxidase labelled competitor) give erroneous measurements in YO bioassays due to the variable presence of endogenous peroxidases. [file Table2.DOCX]

**SUPPLEMENTARY TABLES**

**Table 2.**

LC-MS analysis of ecdysteroids secreted by YO pairs *in vitro*. Blue numbers indicate samples from premolt (D2), red numbers, intermolt YO pairs. YO exposed to 50nM CRZ are denoted by A. Asterisks indicate probable loss of sample. Loq = limit of quantification by multiple reaction monitoring (MRM). Detection limits were: ecdysone, 250ng/ml, (none detected in samples), 3-dehydroecdysone (3dE), 25-deoxyecdysone 10ng/ml. The values obtained are broadly similar to those measured by RIA for molt staged YO in this (and our previous) studies. Note: Enzyme immunoassays (using a variety of different antisera and peroxidase labelled competitor) give erroneous measurements in YO bioassays due to the variable presence of endogenous peroxidases.

| Tube | 25dE | | | 3dE | | |
| --- | --- | --- | --- | --- | --- | --- |
|  | RT (min) | Conc. (ng/ml) | ng per YO | RT  (min) | Conc. (ng/ml) | ng per YO |
| 29 | 8.2 | 114 | 7.18 | 6.8 | 138 | 8.67 |
| 29A | 8.2 | 160 | 10.08 | 6.8 | <loq | 0.00 |
| 30 | 8.2 | 895 | 56.39 | 6.8 | 0 | 0.00 |
| 30A | 8.3 | 0 | 0.00* | 6.7 | 0 | 0.00 |
| 31 | 8.2 | 1243 | 78.32 | 6.8 | <loq | 0.00 |
| 31A | 8.2 | 585 | 36.86 | 6.7 | 0 | 0.00 |
| 32 | 8.2 | 1005 | 63.28 | 6.8 | 0 | 0.00 |
| 32A | 8.3 | 910 | 57.31 | 6.8 | 0 | 0.00 |
| 33 | 8.3 | 0 | 0.00* | 6.8 | 0 | 0.00 |
| 33A | 8.2 | 1757 | 110.66 | 6.8 | <loq | 0.00 |
| 35 | 8.2 | 444 | 27.98 | 6.8 | <loq | 0.00 |
| 35A | 8.2 | 227 | 14.28 | 6.7 | 0 | 0.00 |
| 46 | 8.2 | 130 | 8.21 | 6.8 | <loq | 0.00 |
| 46A | 8.2 | 136 | 8.55 | 6.8 | <loq | 0.00 |
| 47 | 8.3 | 32 | 2.00 | 6.8 | 119 | 7.51 |
| 47A | 8.3 | 85 | 5.37 | 6.8 | 92 | 5.78 |
| 48 | 8.2 | 541 | 34.11 | 6.8 | <loq | 0.00 |
| 48A | 8.2 | 621 | 39.12 | 6.8 | <loq | 0.00 |
| 49 | 8.2 | 53 | 3.37 | 6.8 | 90 | 5.64 |
| 49A | 8.2 | 17 | 1.06 | 6.8 | 0 | 0.00 |
| 50 | 8.2 | 154 | 9.68 | 6.8 | <loq | 0.00 |
| 50A | 8.2 | 97 | 6.09 | 6.8 | <loq | 0.00 |
